# Brexpiprazole vs. Aripiprazole in Patients with Schizophrenia with or Without Comorbid Substance Use Disorder: A 12-Month Real-World Naturalistic Study of Efficacy

**DOI:** 10.3390/brainsci16070744

**Published:** 2026-07-14

**Authors:** Ginevra Lombardozzi, Georgios D. Kotzalidis, Giada Trovini, Emanuela Amici, Alessia Ceccherelli, Giuseppe Albanesi, Valeria Giovanetti, Giovanni Martinotti, Sergio De Filippis

**Affiliations:** 1 Clinica Neuropsichiatrica Villa Von Siebenthal, Via della Madonnina 1, 00040 Roma, Italy; ginevralombardozzi@gmail.com (G.L.); giada.trovini@gmail.com (G.T.); emanuelaamici42@gmail.com (E.A.); giuseppe.albanesi8@gmail.com (G.A.); valeria.giovanetti@yahoo.it (V.G.); sergio.defilippis@me.com (S.D.F.); 2 Section of Psychiatry, Department of Neurosciences, Università Cattolica del Sacro Cuore, Largo Francesco Vito 1, 00168 Rome, Italy; 3 Department of Physiology and Pharmacology “V. Erspamer”, Sapienza University, Piazzale Aldo Moro 5, 00185 Rome, Italy; alessia.ceccherelli@uniroma1.it; 4 Scuola di Medicina e Scienze della Salute, Ospedale SS. Annunziata Chieti, Università degli studi G. D’Annunzio Chieti Pescara, Via dei Vestini, 66100 Chieti, Italy; giovanni.martinotti@unich.it

**Keywords:** aripiprazole, brexpiprazole, partial dopamine D_2_-agonists, schizophrenia spectrum disorders, substance use disorders

## Abstract

**Highlights:**

**What are the main findings?**

- Aripiprazole and brexpiprazole were found to reduce symptoms of schizophrenia.
- The effect of brexpiprazole was particularly prominent in patients with comorbid substance abuse.

**What are the implications of the main findings?**

- Dopamine D_2_ partial agonists efficaciously reduce the symptoms of schizophrenia.
- Brexpiprazole could have some advantage in patients who use substances.

**Abstract:**

**Background**: Schizophrenia with comorbid substance use disorder (SUD) is associated with greater clinical severity, poorer adherence, and worse functional outcomes. Third-generation antipsychotics, through partial dopamine agonism, may represent a useful strategy in this complex population. This study compared the long-term efficacy of brexpiprazole and aripiprazole in patients with schizophrenia, with or without comorbid SUD. **Methods**: Patients (N = 243) with DSM-5/DSM-5-TR schizophrenia orally received 4 mg/day brexpiprazole or 30 mg/day aripiprazole for 12 months in a real-world clinical setting. Psychopathology was assessed at baseline and after 1, 3, 6, and 12 months using the Brief Psychiatric Rating Scale (BPRS) and the Positive And Negative Syndrome Scale (PANSS). Analyses were performed on 217 completers, excluding 26 drop-outs. Outcomes were compared according to drug and presence/absence of comorbid SUD. **Results**: Both brexpiprazole and aripiprazole were associated with psychotic symptom and global psychopathology improvement over 12 months (*p* < 0.01). In patients without SUD, the two treatments showed comparable efficacy. Among patients with comorbid SUD, brexpiprazole showed greater improvement in BPRS and PANSS outcomes than aripiprazole (*p* < 0.01). Treatment response to aripiprazole, but not to brexpiprazole, was diminished by substance use. Brexpiprazole was associated with better tolerability and lower rates of subjective agitation-related discontinuation. **Conclusions**: Both dopamine partial agonists were effective in treating schizophrenia; however, brexpiprazole was superior in patients with comorbid SUD. Findings suggest that brexpiprazole may represent a promising therapeutic option in dual-disorder patients.

## 1. Introduction

Schizophrenia (SCZ) is a severe, chronic, and heterogeneous psychiatric disorder characterized by positive, negative, and cognitive symptoms. Its pathophysiology is primarily associated with dopaminergic dysregulation [1,2], and despite decades of pharmacological progress, its clinical course remains profoundly disabling [3].

Since the introduction of chlorpromazine in 1952 [4], numerous pharmacological agents have been developed for the treatment of SCZ. First-generation antipsychotics (FGAs), primarily D_2_ receptor antagonists, were followed by second-generation antipsychotics (SGAs), which combine D_2_ antagonism with a stronger affinity for serotonin receptors [5]. Around the time of introduction in the market of the chemically heterogeneous SGAs, a molecule with partial agonism at the D_2/3_ dopamine receptors and 5-HT_2A_ serotonin receptors was synthesized in Japan [6,7]. Its antipsychotic properties in animal studies prompted chemists to synthesize agents with similar receptor properties and coined the novel class of dopamine receptor partial agonists [8], which later were termed third-generation antipsychotics (TGAs) [9]. These drugs are characterized by partial agonism at D_2_ and D_3_ receptors and designed to offer efficacy with a lower risk of extrapyramidal and metabolic adverse effects.

Aripiprazole, the first third-generation antipsychotic (TGA), was introduced for SCZ in the United States in 2002, four years before being approved in Japan, its country of origin; it was later followed by brexpiprazole, developed by the same company, in 2015 [10]. Both agents act as D_2_/D_3_ partial agonists with 5-HT_1A_ agonist and 5-HT_2A_ antagonist properties and are regarded as dopamine system stabilizers [11,12,13]. However, they exhibit subtle differences in receptor affinity and activity [14], which likely account for their distinct clinical profiles. Both oral and long-acting injectable formulations of aripiprazole [15] have shown real-world effectiveness and effectiveness in acute schizophrenia episodes [16]; the same holds true for oral brexpiprazole [17], which has also shown improved metabolic profiles and life engagement in patients who were switched from other antipsychotics [18]. Another real-world study found switching to brexpiprazole to have a better safety profile than switching from other antipsychotics to aripiprazole [19].

The clinical need for improved antipsychotic strategies is particularly evident in patients with SCZ and comorbid substance use disorder (SUD), a condition involving nearly half of the SCZ population and that is associated with increased relapse, number of hospitalizations and length of stay at ward, and poorer treatment adherence [20]. SUD comorbidity increases the odds for future hospitalization [21]; hence, it is important to target this comorbidity with appropriate drugs. Since dopaminergic dysregulation also underlies craving and reward-seeking behaviours [22], partial D_2_ agonists may offer therapeutic advantages in this subgroup.

The present naturalistic study aimed to compare the longitudinal real-world effectiveness of brexpiprazole versus aripiprazole in patients with SCZ, with or without comorbid SUD, assessing multiple symptom domains over a 12-month follow-up. For this aim, we included patients with DSM-5/5-TR SCZ and monitored their psychopathology with the use of appropriate rating scales; we compared the two drugs for their effects on the non-comorbid SCZ population and on the comorbid one to detect any differences.

## 2. Materials and Methods

### 2.1. Patients

We carried out a real-world observational study on 243 patients with diagnosis of SCZ who were hospitalized at the Villa Von Siebenthal neuropsychiatric hospital. Recruitment began in January 2022 and ended in October 2022.

*Inclusion criteria.* Eligible patients were adults from 18 to 65 years old and that had a Diagnostic and Statistical Manual (DSM)-5/DSM-5-TR diagnosis of SCZ. A part of the sample had a comorbid diagnosis of one of the following DSM-5/DSM-5-TR SUDs, i.e., cannabis, synthetic cannabinoids, cocaine, amphetamines, opioid, ketamine/phencyclidine or other inhibitors of *N*-methyl-D-aspartate (NMDA) receptors, khat and other alkaloid cathinones, alcohol and polysubstance use disorder. We allowed SUD patients to continue their pharmacological treatment for their specific SUD, for instance, naltrexone, methadone, and buprenorphine on a continuative basis or gabapentinoids, benzodiazepines and other benzodiazepine site agonists not on a continuation basis.

*Exclusion criteria* included age out of the range for inclusion (i.e., <18 years or >65 years), a current comorbid major psychiatric disorder different from SCZ, a high risk of suicide, as assessed with the Columbia-Suicide Severity Risk Scale (C-SSRS) [23], and comorbid severe organic diseases (such as autoimmune or systemic connective tissue diseases, treatment-resistant hypertension, type-1 diabetes or untreated type-2 diabetes, the presence of a metabolic syndrome, severe cardiovascular diseases, and major neurological diseases); furthermore, patients with a history of epilepsy, head injury, electroencephalographic (EEG) abnormalities (current or past), and neurodevelopmental disorders were also excluded; patients with an intelligence quotient (IQ) of <75, as assessed with the Wechsler Adult Intelligence Scale (WAIS), 4th edition [24], those unwilling to participate or those whose legal tutors opposed participation, and inability to sign the informed consent for oneself or, in case of inability, unwillingness/refusal of the legal guardian to sign were also excluded.

Patients who met the inclusion criteria (and did not meet the exclusion criteria) were subsequently told the aims and the methods of the study and provided free, informed consent. The study received approval from the local ethical committee (CE Lazio 2, Rome, Italy; protocol number 331-306-00387 of 27 October 2021). The study adhered to the Principles of Human Rights, as adopted by the World Medical Association at the 18th WMA General Assembly, Helsinki, Finland, June 1964 and subsequently amended by the 64th WMA General Assembly, Fortaleza, Ceará, Brazil, October 2013.

### 2.2. Treatment

In our opinion, TGAs represented the most appropriate treatment option; therefore, we selected aripiprazole and brexpiprazole for this study.

The overall sample of included patients was then divided into two subgroups based on the prescribed treatment: one treated with brexpiprazole, the other with aripiprazole.

Ninety-three patients were treated with brexpiprazole at a daily oral dose of 4 mg. When drug-naïve or drug-free for at least 2 weeks, brexpiprazole was immediately prescribed, following the recommended titration from 1 mg once daily to adjustment to 4 mg once daily. If patients were on treatment with other antipsychotics, they assumed brexpiprazole after a proper cross-titration, as recommended by guidelines and datasheets. Of the 86 completers, 48 patients had a comorbid SUD diagnosis (SCZ-SUD^+^), while 38 had a diagnosis of SCZ without SUD (SCZ-SUD^−^).

**Figure 1:**
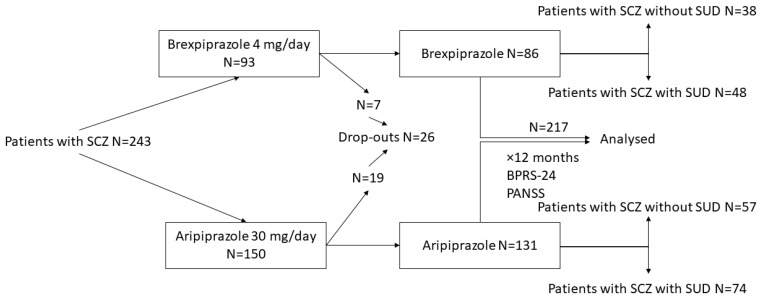
Signal flow diagram of our study design with drug assignment and patient subgroups.

Patients to be treated with 30 mg/day oral aripiprazole (N = 150) were recruited when drug-naïve or drug-free for at least 2 weeks. Aripiprazole was prescribed at first at the recommended starting dose of 10 or 15 mg/day and subsequently titrated to the target dose of 30 mg/day. If patients were on treatment with other antipsychotics, aripiprazole was prescribed with a proper cross-titration from the previous medication; of the 131 completers, 57 patients had a diagnosis of SCZ without SUD, while 74 had comorbid SUD. A signal flow diagram of our study design is shown in Figure 1.

Benzodiazepines or allosteric benzodiazepine receptor modulators were allowed as needed to deal with episodes of psychomotor agitation or insomnia problems. Gabapentin and pregabalin were also allowed on an occasional basis. Patients were allowed to take the drugs used specifically for each SUD, i.e., methadone, buprenorphine and naltrexone. They were not allowed to take other antipsychotics and antidepressants.

The analysis was carried out on 217 patients because 26 dropped out (10.7%). A total of 7 of them were treated with brexpiprazole (3 withdrew at their own request due to reported subjective agitation and 4 were lost to follow-up) and 19 with aripiprazole (13 withdrew at their own request due to reported subjective agitation and 6 were lost at follow-up).

### 2.3. Study Assessments

Completers were followed for 12 months and were regularly assessed. Psychometric scales were used to assess their psychopathology at pretreatment baseline, after 1 month, and after 3, 6, and 12 months from recruitment.

The diagnosis of SCZ was made with the SCID-5-CV [25] by skilled psychiatrists who also investigated the presence of comorbid SUD in these patients (cannabis, synthetic cannabinoids, cocaine, amphetamines, opioid, ketamine/phencyclidine or other NMDA receptor inhibitors, khat and other cathinone alkaloids, alcohol and polysubstance use disorder).

Psychopathology was evaluated and rated with the 24-item expanded 4.0 version of the Brief Psychiatric Rating Scale (BPRS) [26], Italian version [27], and the Positive And Negative Syndrome Scale (PANSS) [28].

### 2.4. Statistical Analysis

Descriptive statistics were first calculated, with categorical variables expressed as count and percentage and continuous variables reported as mean, standard deviation, median, minimum and maximum. To assess the sample distribution, normality was checked. Skewness and kurtosis values were found to be within the acceptable range confirming a normal distribution. Demographic and baseline clinical characteristics were compared between the two study groups using analysis of variance (ANOVA) for continuous variables and the chi square test for categorical variables. If demographic and baseline clinical characteristics differed between two groups, analysis of covariance (ANCOVA) was conducted using these characteristics as covariates. Analysis of covariance (ANCOVA) was used to compare the change in clinical outcomes (BPRS total score, PANSS total score, PANSS positive scale, PANSS negative scale, and PANSS general psychopathology scale) from baseline including the treatment group and the use of substances as factors and the baseline scale value as a covariate. The significance level was set at 0.05, and Bonferroni’s correction was performed when testing for multiple dependent variables. All tests were two-sided. For all statistical analyses, the Statistical Package for Social Science (SPSS) version 29.0 (IBM, Armonk, NY, USA) was used.

## 3. Results

Our sample consisted of 243 patients with SCZ, 93 (38.3%) on brexpiprazole and 150 (61.7%) on aripiprazole. Patients’ ages ranged from 18 to 65 years (mean 38.6, standard deviation (SD) = 16.44). The demographic and baseline characteristics of the sample are shown in Table 1. Of the 243 patients who were included in the sample, 217 (86 in the brexpiprazole group; 131 in the aripiprazole group) were analysed because 26 (10.7%) patients dropped out from the study. The complete analysis is shown in the Supplementary Materials (Figures S1–S9 and Table S1).

### 3.1. BPRS Total Score

**Figure 2:**
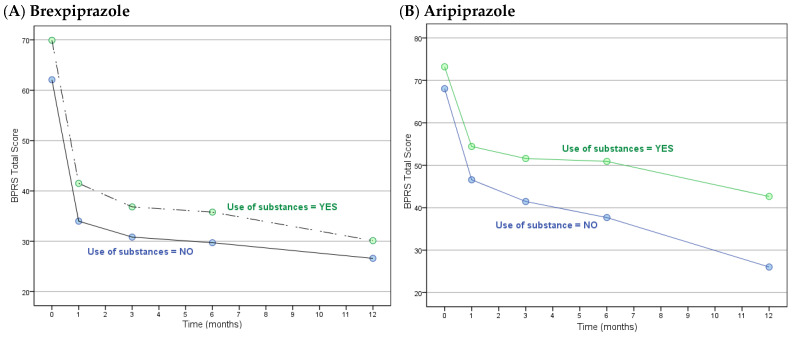
BPRS total score longitudinal analysis from baseline to the 12-month follow-up.

Brexpiprazole group: At baseline, the non-SUD group (N = 38) scored 62.1 ± 13.81 on the BPRS total score, while the SUD group (N = 48) scored 69.9 ± 19.33 (independent samples *t*-test *p* = 0.038), and at endpoint (assessment at 12 months), they scored 26.6 ± 2.88 and 30.1 ± 6.53, respectively (independent samples *t*-test *p* = 0.003). Both groups showed significant decrements from baseline to endpoint (paired sample *t*-test *p* < 0.001). The BPRS total score longitudinal analysis is shown in Figure 2A.

Aripiprazole group: At baseline, the non-SUD group (N = 57) scored 68.1 ± 13.20 on the BPRS total score, while the SUD group (N = 74) scored 73.2 ± 20.62 (independent samples *t*-test *p* = 0.103), and at endpoint (assessment at 12 months), they scored 26.0 ± 8.99 and 42.7 ± 19.46, respectively (independent samples *t*-test *p* < 0.001). Both groups showed significant decrements from baseline to endpoint (paired sample *t*-test *p* < 0.001). The BPRS score longitudinal analysis is shown in Figure 2B.

A significantly greater BPRS total score decrease was observed in the group of patients treated with brexpiprazole compared to those treated with aripiprazole (ANCOVA *p*-value = 0.003; brexpiprazole −39.8 vs. aripiprazole −34.3; Table 2).

**Figure 3:**
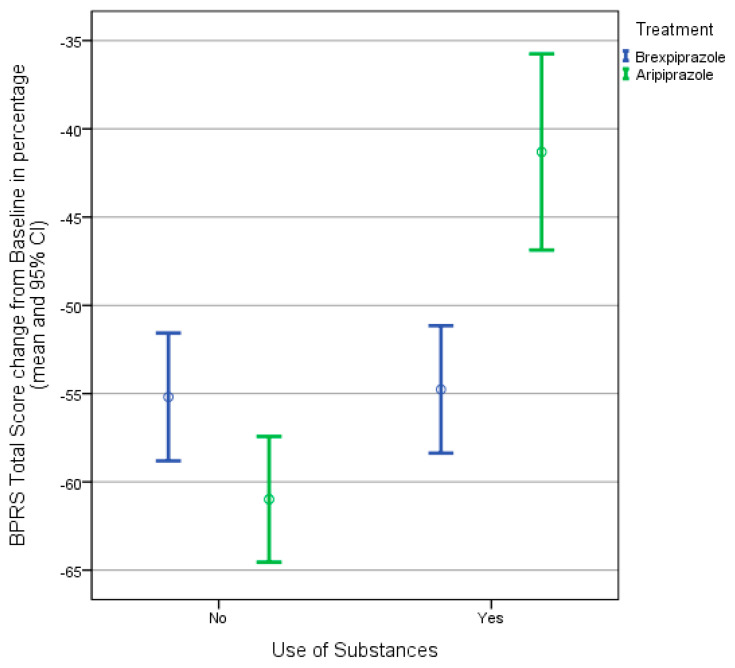
BPRS total score change from baseline analysis (up to the 12-month follow-up). Brexpiprazole, blue; aripiprazole, green.

The BPRS total score decrease was statistically different when comparing non-SUD versus SUD patients (ANCOVA *p*-value < 0.001; Figure 3). We found an interaction of time × SUD (presence/absence), with *p* < 0.001, in which symptoms improved in both conditions over time.

### 3.2. PANSS Total Score

**Figure 4:**
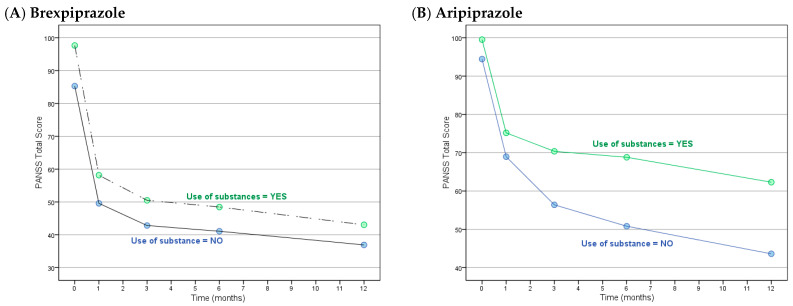
PANSS total score longitudinal analysis from baseline to the 12-month follow-up.

Brexpiprazole group: At baseline, the non-SUD group (N = 38) scored 85.3 ± 14.94 on the PANSS total score, while the SUD group (N = 48) scored 97.7 ± 22.23 (independent samples *t*-test *p* = 0.004), and at endpoint (assessment at 12 months), they scored 36.9 ± 6.36 and 43.0 ± 11.95, respectively (independent samples *t*-test *p* = 0.005). Both groups showed significant decrements from baseline to endpoint (paired sample *t*-test *p* < 0.001). The BPRS total score longitudinal analysis is shown in Figure 4A.

Aripiprazole group: At baseline, the non-SUD group scored 94.4 ± 16.25 on the PANSS total score, while the SUD group scored 99.5 ± 24.71 (independent samples *t*-test *p* = 0.181), and at endpoint (assessment at 12 months), they scored 43.6 ± 11.99 and 62.3 ± 25.0, respectively (independent samples *t*-test *p* < 0.001). Both groups showed significant decrements from baseline to endpoint (paired sample *t*-test *p* < 0.001). The BPRS score longitudinal analysis is shown in Figure 4B.

A significantly greater decrease in PANSS total scores was observed in the group of patients treated with brexpiprazole compared to the one treated with aripiprazole (ANCOVA *p*-value < 0.001; brexpiprazole −54.1 vs. aripiprazole −41.7; Table 3).

**Figure 5:**
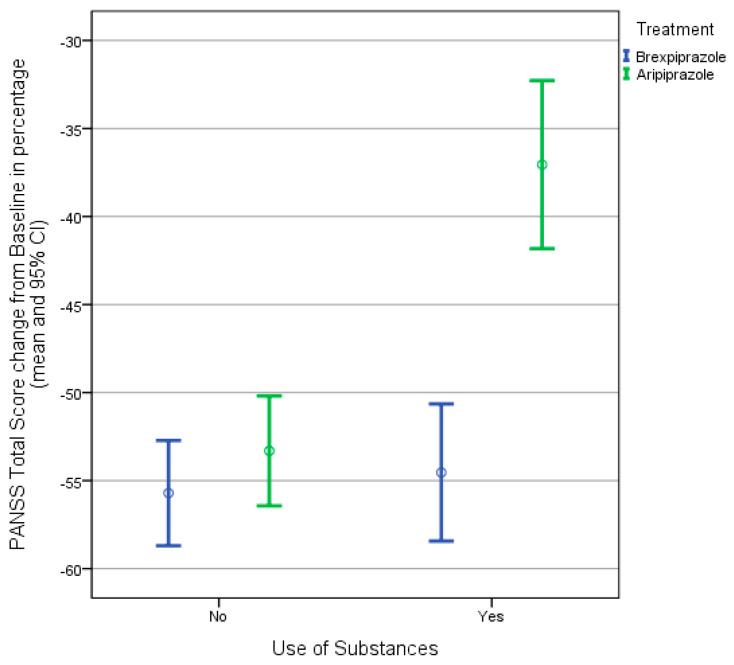
PANSS total score change from baseline analysis (to the 12-month endpoint). Brexpiprazole, blue; aripiprazole, green.

When comparing SCZ-SUD^−^ vs. SCZ-SUD^+^ patients, PANSS total scores decreased significantly more in the former (ANCOVA *p*-value < 0.001; Figure 5). We found an interaction of time × SUD (presence/absence), with *p* < 0.001, in which symptoms improve in both conditions over time.

In the brexpiprazole group, comparing the five timepoints for each subscale of the PANSS, we found a main effect of time for the negative symptoms and the general psychopathology subscales (% 12-month change from baseline −55.9% and −55.5%, respectively; Table 4).

In the aripiprazole group, comparing the five timepoints for each subscale of the PANSS, we found a main effect of time for the negative symptoms subscale and the general psychopathology subscale (% 12-month change from baseline −45.6% and −41.7%, respectively; Table 5).

Percentage changes from baseline for both PANSS subscales were stronger in the brexpiprazole group compared to aripiprazole.

### 3.3. Concomitant Treatments in the Sample Other than Brexpiprazole and Aripiprazole

The drug classes of allowed substances during the course of the 1-year follow-up are shown in Table 6. The results do not distinguish between SUD and non-SUD status.

### 3.4. Distribution of Substances Used in the SCZ-SUD^+^ Samples for Aripiprazole (N = 74) and Brexpiprazole (N = 48)

Table 7 shows the distribution of SUDs in the two samples.

Since some SUDs were underrepresented, it was not possible to perform statistical analyses separately for each substance used.

## 4. Discussion

This 12-month naturalistic study compared brexpiprazole and aripiprazole, two dopamine–serotonin partial agonist TGAs in patients with schizophrenia with or without comorbid SUD.

The overall results showed that both medications were effective throughout the time of treatment in positive psychotic and negative symptoms and In terms of global psychopathology, which also includes affective symptoms, behavioural dysregulation and disorientation.

In both PANSS and BPRS, a significant decrease in scores (*p*-values < 0.01) was observed at the 12-month time point. However, when stratifying by substance use (yes/no), a different pattern emerged: a substantial balance between the two treatments was observed in the cohort of patients who did not use substances, whereas a clear advantage (significant, *p*-value < 0.01) of treatment with brexpiprazole was observed in the cohort of patients who used substances. This was adjusted for the baseline values of the scales.

In patients treated with aripiprazole, efficacy significantly differed according to substance use status, with greater efficacy observed in patients who did not use substances. This difference was not observed in patients treated with brexpiprazole. The differences between aripiprazole and brexpiprazole in their receptor binding properties may have accounted for this difference and for brexpiprazole overcoming the relative treatment resistance conferred by coexistence of SCZ with SUD.

The clinical significance of these findings is strengthened by the neurobiological rationale that distinguishes the pharmacodynamics of the two molecules.

Treating schizophrenia remains a major clinical challenge, and new antipsychotic agents have been developed over the years to address its multiple therapeutic and tolerability issues. Within the class of TGAs, aripiprazole and its derivative brexpiprazole share a similar pharmacological framework but exhibit subtle yet meaningful pharmacodynamic differences that may account for variations in their clinical profiles and therapeutic applications [10].

To contextualize these findings, it is useful to briefly outline current antipsychotic pharmacotherapy from FGAs to SGAs and, more recently, to TGAs.

Medications to treat schizophrenia can be divided into three groups, i.e., FGAs, SGAs, TGAs, and others. FGAs mainly act on positive symptoms as antagonists for the dopamine type 2 (D_2_) receptor; for the same reason, they are frequently associated with extrapyramidal symptoms (EPSs), increased levels of prolactin and worsening of negative symptoms.

SGAs exhibit higher affinity for serotonin receptors (5-HT) than D_2_ receptors, with some exceptions, and they also exhibit actions on muscarinic cholinergic (M_1_, M_3_ and M_4_), histamine (H_1_), and alpha-adrenergic receptors (α_1_ and α_2_). They are less associated with EPS than FGAs, but they may lead to remarkable metabolic dysregulation (weight gain, diabetes, hyperlipidaemia, QT prolongation) [29]. TGAs are the latest class of molecules that are distinguished by their partial agonist action on dopamine D_2_ receptors; they are meant to stabilize the levels of dopamine with a reduction in positive symptoms, improvement in negative symptoms and low risk of extrapyramidal syndrome onset and weight gain. TGAs were intended to tackle the task of making drugs available that increase the availability of dopamine in the cortex while limiting its action in the limbic system. It has been shown that D_2_ dopamine receptor blockade is efficacious in controlling positive symptoms but is of little help in the control of negative symptoms, and if anything, it worsened them [30].

Aripiprazole and brexpiprazole both belong to TGAs. Aripiprazole was developed and approved in 2002 by the FDA as a treatment for schizophrenia (SCZ) and in Italy by the Italian Drug Agency (AIFA) in 2004; later, it was approved for other indications, including bipolar disorder and major depressive disorder as an adjunctive therapy [31]. Later, this pharmacological category was enriched at first by the development of cariprazine and then by the development, by the original developers of aripiprazole, of brexpiprazole [10]. Both received approval for SCZ in 2015 [32,33].

Compared to aripiprazole, brexpiprazole shows a lower intrinsic D_2_ activity [34], meaning it acts less as a dopaminergic stimulator, potentially reducing the risk of activation, agitation and akathisia while maintaining antipsychotic effects, thus having a better overall tolerability profile.

In addition to this, brexpiprazole exhibits higher binding affinity for 5-HT_1A_, 5-HT_2A_ and α_1B_ receptors. These features are believed to enable a better mitigation of extrapyramidal symptoms, akathisia, insomnia, restlessness, and nausea, given that 5-HT_2A_ antagonism, 5-HT_1A_ partial agonism, and α_1B_ antagonism are known to buffer D_2_ blockade within striatal pathways [35,36,37].

The high-affinity antagonism at the 5-HT_2A_ and 5-HT_7_ receptors enhances serotonergic transmission in cortical and limbic circuits, facilitating antidepressant and anxiolytic outcomes through disinhibition of dopaminergic and glutamatergic pathways [11,36,37,38,39]. Furthermore, brexpiprazole’s stronger inhibition of serotonin 5-HT_7_ receptors than aripiprazole [34,35,36,37] increases the likelihood of brexpiprazole inducing positive cognitive effects [39,40,41,42,43]. The positive effects of serotonin 5-HT_7_ receptor blockade on cognition are likely mediated through a glutamatergic function normalization [41,44].

Simultaneously, α_1_-adrenergic receptor antagonism contributes to the reduction in hyperarousal and anxiety-related autonomic activation while also improving sleep and emotional regulation [45,46]. Brexpiprazole is a stronger α_1B_ and α_2C_ adrenoceptor blocker than aripiprazole [34].

Impulsiveness in patients with schizophrenia has been linked to dysregulation of dopaminergic signalling within the fronto-striatal circuitry, particularly involving the orbitofrontal cortex and the nucleus accumbens [47]. Excessive D_2_ receptor blockade in these regions may impair top-down inhibitory control, leading to behavioural disinhibition and increased impulsive responding. A lower intrinsic D_2_ activity, resulting in lower dopaminergic stimulation, and stronger serotonergic modulation (notably 5-HT_1A_ agonism and 5-HT_2A_ antagonism) could result in a more balanced effect on impulsivity and emotional regulation compared with aripiprazole [46,48].

These mechanisms align with the observed improvements not only in positive symptoms but also in negative and affective dimensions: they may underlie brexpiprazole’s antidepressant effects and its beneficial effects on emotional regulation in our population. Brexpiprazole is used in treatment-resistant depression and bipolar disorder [49,50], conditions in which emotional dysregulation is prominent. Emotional dysregulation is frequently found in bipolar disorder [51,52,53] and constitutes a primary driver in treatment-resistant depression [54].

In our study, we aimed to focus on comorbidity with SUD. According to the findings of an Italian survey, the clinical management of schizophrenia comorbid with substance use is perceived by clinicians as increasingly complex due both to the heterogeneity of the substances involved, to the deterioration in interpersonal and social functioning that accompanies dual disorder, and to the increased prevalence of SUDs in the general population [55].

This population represents a major therapeutic challenge, as traditional antipsychotic treatments may exacerbate certain clinical features, increase the risk of relapse into substance use, and compromise treatment adherence. In fact, such drugs, being potently antidopaminergic, may trigger hypodopaminergia that can provide the basis for substance craving, thus boosting non-adherence and relapse [56].

These functions are crucial in individuals with schizophrenia and SUD, where dopaminergic dysregulation extends into reward-seeking behaviour, craving intensity and vulnerability to relapse. The robust reduction in substance craving observed in this subgroup suggests that brexpiprazole may better modulate the motivational circuitry involving the ventral striatum, prefrontal cortex, and amygdala—regions where dopamine–serotonin interactions determine the balance between reward anticipation and inhibitory control [57]. Neuroimaging evidence showed that schizophrenia comorbidity amplified or uniquely modified the neurobiological alterations associated with SUDs, highlighting distinct effects across dopaminergic, striatal and prefrontal circuits [58].

Usually, comorbid SUD hampers treatment efficacy [59] since it is associated with more severe psychotic symptoms and reduced treatment adherence. In a large real-world cohort, Burrer et al. [60] showed that comorbid substance use in schizophrenia was associated with increased hospitalization frequency and distinct patterns of length of stay, underscoring the negative impact of single and multiple SUDs on the course of illness. Furthermore, antipsychotic-medication-induced D_2_ blockade tends to interfere with the reward circuitry, leading to increased odds for drug-seeking, behavioural dysregulation and relapse [61,62].

Using electronic health record data, Patel et al. [63] demonstrated that comorbid SUDs correlated with greater illness severity, reduced treatment persistence, and altered hospitalization patterns in schizophrenia, indicating a consistently poorer clinical trajectory in the SUD population.

Neyra et al. [64] emphasized that SCZ with comorbid SUD requires integrated care models and that partial dopamine agonists—including aripiprazole, cariprazine, and brexpiprazole—show favourable profiles in controlling psychotic symptoms and substance-related behaviours. Furthermore, a systematic review of neuroimaging studies found SUD to amplify the effects of SCZ, presumably through an enhancement of striatal and prefrontal dopaminergic alterations [58], alterations which may be central to the neurobiology of both SCZ and SUD [15].

In our study, we decided to compare aripiprazole to brexpiprazole, which have already been found to constitute good options in treatment of schizophrenia with SUD.

Partial dopamine D_2/3_ agonists—especially long-acting injectable (LAI) aripiprazole—were found to retain clinical effectiveness even in the presence of cannabis use disorder, with the LAI formulation producing the strongest improvements in global psychopathology and craving, supporting these agents as a well-tolerated and advantageous therapeutic option for SCZ spectrum disorders with comorbid substance use [65].

In their updated systematic review, Santorelli et al. [66] reported that aripiprazole—both in its oral formulation and as an LAI—consistently improved psychotic symptoms and produced a significant reduction in craving among individuals with SCZ and comorbid SUD.

Brexpiprazole has already proven to be effective in reducing psychotic symptoms and improving global clinical picture in an observational study of patients affected by schizophrenia with no differences in the SUD subpopulation, showing a marked reduction in craving [67].

In a randomized study where patients with a diagnosis of SCZ and co-occurring SUD were randomized in a group that switched to brexpiprazole and a group that were maintained on their prescribed antipsychotics, patients treated with brexpiprazole showed a marked decrease in craving and in money spent to buy the substance [68].

An Italian multicentre, real-world, prospective study of brexpiprazole specifically focusing on a comorbid SCZ-SUD population reported positive effects of the drug on craving and global functioning [69]. The usefulness of brexpiprazole in the SCZ-SUD^+^ population is further strengthened by the findings of a recent narrative review of real-world evidence (2020–2026), which confirmed its favourable tolerability and efficacy profiles in this population [70].

The improvement profile observed with brexpiprazole, regarding efficacy on core symptoms of SCZ, action on the mood and affective symptoms associated with craving in the SUD-comorbid population, and less extrapyramidal symptoms, embraces domains that strongly influence real-world functioning, quality of life and perception of disability [71]. This is clinically meaningful because these domains are powerful determinants of adherence, therapeutic alliance, and autonomy [72]. Among the drugs that have been used to treat SCZ-SUD^+^ patients and found to reduce craving, there is the atypical lurasidone (it blocks D_2_ dopamine receptors more than 5-HT_2A_ serotonin receptors while being even stronger at 5-HT_7_ serotonin receptors [73]), which proved to reduce craving and improve quality of life of “dual” patients with alcohol use disorder in an open-label study [74]. We did not use lurasidone as a comparison drug because it is not a partial dopamine D_2/3_ agonist, but we also did not compare the two D_2/3_ agonists with the other D_2/3_ agonist, cariprazine, despite the fact that this drug was shown to decrease symptoms of SCZ and cannabis use in dual SCZ and cannabis use disorder patients in one 6-month open study, which did not specifically address craving [75]. We will deal with this weakness of our study later, in the Limitations section. Overall, the practice of administering atypical antipsychotics to patients with comorbid SCZ and SUD is widely consolidated [76], which legitimized our real-world study.

Together, these findings suggest that brexpiprazole may offer a clinically meaningful advantage over aripiprazole in patients with SCZ, particularly in the presence of comorbid SUD, which can represent a barrier to treatment. However, caution is needed in interpreting our results, since the aripiprazole (*N* = 131) and brexpiprazole (*N* = 86) groups were imbalanced, and this might have affected the results. At any rate, it was not possible to balance the two drugs, since aripiprazole is used since sometimes and brexpiprazole is a relatively recent introduction in the antipsychotic market. This has skewed the sample towards aripiprazole, and this was expected for a real-world observational study such as ours.

### Limitations

This study has several limitations. Its naturalistic design may hinder causal inference through unmeasured confounding factors, such as concomitant medications. Furthermore, although the sample was relatively large for a single-centre study, it may not apply to other populations; hence, it needs replication in larger, multicentre samples. The sample was relatively imbalanced towards aripiprazole (1.52:1); this was also reflected in the SCZ-SUD^+^ subgroups (1.54:1). However, the percentages of SCZ-SUD^+^ patients in the two groups were similar (56.49% in the aripiprazole group and 55.81% in the brexpiprazole group). Another limitation involves the lack of comparison with the third TGA, i.e., cariprazine, and no use of placebo. However, our study was a real-word naturalistic study, so this design was the only one we could afford. Moreover, in our analysis, we did not stratify outcomes by gender, duration of illness, or treatment history (drug-naïve vs. switched patients). Finally, the definition of SUD was clinically consistent but did not differentiate specific substance types, potentially masking differential effects across substance categories. It should be kept in mind that different substances are associated with different clinical presentations, psychosis profiles and clinical trajectories [77,78]. The heterogeneity of presentations is further enhanced by the use of novel psychoactive substances [71], but this was not specifically focused upon in our study. This heterogeneity of clinical features might have been reflected in the increased clinical variability we observed in our two SCZ-SUD^+^ groups. We also did not compare the effects of the two drugs on craving and impulsiveness in this study, as already performed by others [61,62]; however, this will be the subject of a further study, where some of the confounders will be addressed. Last, we did not analyze our data according to the duration of untreated psychosis, age at onset of SCZ and SUD, the duration of drug treatment, and the smoking habits of our patients. The use of tobacco may affect blood levels of drugs like clozapine and olanzapine, but such drugs were not used here, so we did not need to perform therapeutic drug monitoring, which is another limitation, since both aripiprazole and brexpiprazole have therapeutic windows for their serum levels [46,79,80]; the two drugs we used, i.e., aripiprazole and brexpiprazole, are not affected by tobacco use [81].

## 5. Conclusions

TGAs, characterized by partial dopamine agonism and receptor-selective profiles, represent a rational pharmacological strategy for managing SCZ. Aripiprazole and its derivative brexpiprazole proved here to both be effective, although their distinct receptor-binding properties may account for subtle differences in their clinical effects. In this study, TGAs were associated with significant improvements in positive and negative symptoms of SCZ over the 12-month follow-up. However, patients treated with brexpiprazole showed a greater reduction in global psychopathology, particularly among those with comorbid SUD. Furthermore, brexpiprazole had its effectiveness unaffected even in the presence of comorbid SUD, a condition that often complicates treatment response and adherence. Further controlled studies are needed to confirm these observations and to clarify the potential advantages of brexpiprazole in the clinically challenging SCZ-SUD^+^ population.

## Figures and Tables

**Table 1:** Demographics and baseline characteristics of the study population (full analysis set, N = 243).

	Brexpiprazole (N = 93)	Aripiprazole (N = 150)	*p*-Value
Age (years)			
mean (SD)	39.3 (14.24)	38.2 (17.70)	0.599
median (min–max)	38.0 (18–65)	29.0 (18–65)	
Gender, N (%)			
male	57 (61.3)	98 (65.3)	0.524
female	36 (38.7)	52 (34.7)	
BPRS Total Score			
mean (SD)	65.7 (17.18)	72.1 (17.98)	0.007
median (min–max)	64.0 (38–115)	70.0 (5–115)	
PANSS Positive Items Score			
mean (SD)	16.3 (8.87)	18.8 (9.03)	0.036
median (min–max)	14.0 (7–39)	17.5 (5–39)	
PANSS Negative Items Score			
mean (SD)	23.1 (6.36)	26.2 (6.0)	<0.001
median (min–max)	25.0 (7–34)	27.0 (5–38)	
PANSS General Psychopathology Items Score			
mean (SD)	51.7 (11.51)	53.5 (11.01)	0.236
median (min–max)	53.0 (25–79)	55.0 (5–77)	
PANSS TOTAL Score			
mean (SD)	91.0 (20.34)	98.3 (20.95)	0.008
median (min–max)	91.0 (49–149)	98.5 (5–149)	
Attrition			
Drop-out patients, N (%)	7 (7.5)	19 (12.7)	-
Analysis population, N (%)	86 (92.5)	131 (87.3)	-

Abbreviations: BPRS, Brief Psychiatric Rating Scale (expanded, 24-item 4.0 version); PANSS, Positive And Negative Syndrome Scale; SD, standard deviation.

**Table 2:** BPRS total score ANCOVA: brexpiprazole vs. aripiprazole.

**EMMs**					
**Dependent Variable: BPRS Total Score Month 12 Change from Baseline.**					
**Treatment**	$\bar{x}$	**S.E.M.**	**95%C.I.**		
			**Lower**	**Upper**	
Brexpiprazole	−39.793	1.437	−42.625	−36.960	
Aripiprazole	−34.296	1.162	−36.588	−32.005	
**Pairwise Comparisons**					
Dependent Variable: BPRS Total Score Month 12 change from Baseline.					
	$\bar{x}\Delta$	S.E.M.	*p*	95%C.I. for Difference	
				Lower	Upper
Brexpiprazole–Aripiprazole	−5.497	1.855	**0.003**	−9.153	−1.840

Abbreviations: BPRS, Brief Psychiatric Rating Scale; EMMs, estimated marginal means; *p*, statistical significance probability; S.E.M., standard error mean; $\bar{x}$, mean; $\bar{x}\Delta$, mean difference; 95%C.I., 95 percent confidence interval. **Bold characters** in Table boxes, significant results.

**Table 3:** PANSS total score ANCOVA: brexpiprazole vs. aripiprazole.

**EMMs**						
**Dependent Variable: PANSS Total Score Month 12 Change from Baseline.**						
**Treatment**	$\bar{x}$		**S.E.M.**		**95%C.I.**	
					**Lower**	**Upper**
Brexpiprazole	−54.099		1.614		−57.281	−50.918
Aripiprazole	−41.668		1.306		−44.241	−39.094
**Pairwise Comparisons**						
Dependent Variable: PANSS Total Score Month 12 Change from Baseline.						
	$\bar{x}\Delta$	S.E.M.		*p*	95%C.I. for Difference	
					Lower	Upper
Brexpiprazole–Aripiprazole	−12.432	2.083		**0.000**	−16.538	−8.325

Abbreviations: EMMs, estimated marginal means; p, statistical significance probability; PANSS, Positive And Negative Syndrome Scale; S.E.M., standard error mean; $\bar{x}$, mean; $\bar{x}\Delta$, mean difference; 95%C.I., 95 percent confidence interval. **Bold characters** in Table boxes, significant results.

**Table 4:** Brexpiprazole: percentage PANSS subscale change from baseline to the 12-month endpoint.

Substance Use		% Change from Baseline		
		PANSS-P	PANSS-N	PANSS-GP
No	$\bar{x}$	−34.12	−55.91	−57.43
	N	38	38	38
	S.D.	22.297	19.806	10.541
Yes	$\bar{x}$	−45.61	−55.93	−53.91
	N	48	48	48
	S.D.	24.178	16.764	14.814
Total	$\bar{x}$	−40.53	−55.92	−55.47
	N	86	86	86
	S.D.	23.928	18.059	13.146

Abbreviations: PANSS, Positive And Negative Syndrome Scale; PANSS-GP, PANSS general psychopathology dimension; PANSS-N, PANSS negative symptoms; PANSS-P, PANSS positive symptoms; S.D., standard deviation; $\bar{x}$, mean.

**Table 5:** Aripiprazole: percentage PANSS subscale change from baseline to the 12-month endpoint.

Substance Use		Change from Baseline in Percentage (%)		
		PANSS-P	PANSS-N	PANSS-GP
No	$\bar{x}$	−35.66	−54.62	−51.04
	N	57	57	57
	S.D.	18.059	12.769	12.653
Yes	$\bar{x}$	−36.30	−38.69	−34.49
	N	74	74	74
	S.D.	22.161	20.618	20.853
Total	$\bar{x}$	−36.02	−45.62	−41.69
	N	131	131	131
	S.D.	20.405	19.282	19.519

Abbreviations: PANSS, Positive And Negative Syndrome Scale; PANSS-GP, PANSS general psychopathology dimension; PANSS-N, PANSS negative symptoms; PANSS-P, PANSS positive symptoms; S.D., standard deviation; $\bar{x}$, mean.

**Table 6:** Concomitant treatments with other drug classes in the aripiprazole (*N* = 131) and brexpiprazole (*N* = 86) groups.

Concomitant Treatment	Aripiprazole, *N* (%)	Brexpiprazole *N* (%)
Mood Stabilizers	83 (63.37%)	62 (72.10%)
Antidepressants	29 (22.14%)	35 (40.70%)
Benzodiazepines	83 (63.36%)	20 (28.58%)
Other Antipsychotics	79 (60.31%)	73 (84.88%)

**Table 7:** SUDs in the aripiprazole (*N* = 74) and brexpiprazole (*N* = 48) groups.

Substance Used	Aripiprazole—Comorbid SUD *N* (%)	Brexpiprazole—Comorbid SUD *N* (%)
Cannabis	12 (16.22%)	14 (29.17%)
Alcohol	1 (1.35%)	5 (10.42%)
Cocaine	3 (4.05%)	5 (10.42%)
Polysubstance	58 (78.38%)	24 (50%)

## Data Availability

Anonymized data supporting the conclusions of this article will be made available by the senior author upon reasonable request. Data are not publicly available due to privacy reasons.

## Supplementary Materials

The following supporting information can be downloaded at: https://www.mdpi.com/article/10.3390/brainsci16070744/s1, Figure S1. Brexpiprazole group. SUD^+^ and SUD^−^ subgroups compared for BPRS total scores; Figure S2. Brexpiprazole group. SUD^+^ and SUD^−^ subgroups compared for PANSS total scores; Figure S3. Aripiprazole group. SUD^+^ and SUD^−^ subgroups compared for BPRS total scores; Figure S4. Aripiprazole group. SUD^+^ and SUD^−^ subgroups compared for PANSS total scores; Figure S5. SCZ-SUD^−^ and SUD^+^ brexpiprazole and aripiprazole groups compared for % changes from baseline in BPRS total scores; Figure S6. SCZ-SUD^−^ and SUD^+^ brexpiprazole and aripiprazole groups compared for % changes from baseline in PANSS total scores; Figure S7. SCZ-SUD^−^ and SUD^+^ brexpiprazole and aripiprazole groups compared for % changes from baseline in PANSS positive scores; Figure S8. SCZ-SUD^−^ and SUD^+^ brexpiprazole and aripiprazole groups compared for % changes from baseline in PANSS negative scores; Figure S9. SCZ-SUD^−^ and SUD^+^ brexpiprazole and aripiprazole groups compared for % changes from baseline in PANSS general psychopathology scores; Table S1. Analysis: database brexpiprazole vs. aripiprazole. STROBE statement.

## Abbreviations

The following abbreviations are used in this manuscript:

AIFA	Agenzia Italiana del Farmaco (Italian Drug Agency)
BPRS	Brief Psychiatric Rating Scale, Expanded version 4.0
C-SSRS	Columbia-Suicide Severity Risk Scale
DSM-5/-5-TR	Diagnostic and Statistical Manual of Mental Disorders, 5th edition/-Text Revision
EEG	Electroencephalographic, Electroencephalography, Electroencephalogram
FGA(s)	First-Generation Antipsychotic(s)
IQ	Intelligence Quotient
NMDA	*N*-methyl-D-aspartate
PANSS	Positive And Negative Syndrome Scale
SCID-5-CV	Structured Clinical Interview for DSM-5^®^ Disorders, Clinician Version
SCZ-SUD^−^	Patients with Schizophrenia without Concomitant Substance Use Disorder
SCZ-SUD^+^	Patients with Schizophrenia Comorbid with Substance Use Disorder(s)
SD	Standard Deviation
SGA(s)	Second-Generation Antipsychotic(s)
SUD	Substance Use Disorder
TGA(s)	Third-Generation Antipsychotic(s)
WAIS	Wechsler Adult Intelligence Scale
WMA	World Medical Association
