# Supplementary material for: Brexpiprazole vs. Aripiprazole in Patients with Schizophrenia with or Without Comorbid Substance Use Disorder: A 12-Month Real-World Naturalistic Study of Efficacy
Source: Brain Sci. 2026 Jul 14;16(7):744. doi: 10.3390/brainsci16070744 (PMC13406489; doi:10.3390/brainsci16070744)
Supplement: Supplementary file 1 [file brainsci-16-00744-s001.zip › brainsci-4375145-supplementary.pdf]

brain sciences

MDPI

Supplement for:

Brexpiprazole *vs*. Aripiprazole in Patients with Schizophrenia With or Without Comorbid Substance Use Disorder: A 12-month Real-World Naturalistic Study of Efficacy

Ginevra Lombardozzi [1], Georgios D. Kotzalidis [1,2*], Giada Trovini [1], Emanuela Amici [1], Alessia Ceccherelli [3], Giuseppe Albanesi [1], Valeria Giovanetti [1], Giovanni Martinotti [4], Sergio De Filippis [1]

[1] Clinica Neuropsichiatrica Villa Von Siebenthal, Via della Madonnina 1, 00040 Roma, Italy; ginevralombardozzi@gmail.com; giada.trovini@gmail.com; emanuelaamici42@gmail.com; valeria.giovanetti@yahoo.it; giuseppe.albanesi8@gmail.com; sergio.defilippis@me.com
[2] Section of Psychiatry, Department of Neurosciences, Università Cattolica del Sacro Cuore, Largo Francesco Vito 1, 00168 Rome, Italy; giorgio.kotzalidis@gmail.com
[3] NESMOS Department, Faculty of Medicine and Psychology, Sapienza University of Rome, Via di Grottarossa 1035-1039, 00189 Rome, Italy; alessia.ceccherelli@uniroma1.it
[4] Scuola di Medicina e Scienze della Salute, Ospedale SS. Annunziata Chieti, Università degli studi G. D'Annunzio Chieti Pescara, Via dei Vestini, 31 - 66100 Chieti, Italy; giovanni.martinotti@unich.it
* Correspondence: giorgio.kotzalidis@gmail.com

Index

	Page
Supplementary Table S1. Analysis: Database Brexpiprazole *vs*. Aripiprazole	S2-S18
Supplementary Figure S1	S3
Supplementary Figure S2	S4
Supplementary Figure S3	S6
Supplementary Figure S4	S7
Supplementary Figure S5	S14
Supplementary Figure S6	S15
Supplementary Figure S7	S16
Supplementary Figure S8	S17
Supplementary Figure S9	S18
STROBE Statement	S19-S21
Literature for the STROBE Statement	S22

Supplementary Table S1. Analysis: Database Brexpiprazole *vs*. Aripiprazole

Version: Draft 4.0

Date: 27 February, 2026

Demographics and Baseline Characteristics of the study population. (Full Analysis Set, N = 243).

	Brexipiprazole (N = 93)	Aripiprazole (N = 150)	p-value
Age (years)			
mean (SD)	39.3 (14.24)	38.2 (17.70)	0.599
median (min – max)	38.0 (18 – 65)	29.0 (18 – 65)	
Gender, N (%)			
males	57 (61.3)	98 (65.3)	0.524
females	36 (38.7)	52 (34.7)	
BPRS Total Score			
mean (SD)	65.7 (17.18)	72.1 (17.98)	0.007
median (min – max)	64.0 (38 – 115)	70.0 (5 – 115)	
PANSS Positive Items Score			
mean (SD)	16.3 (8.87)	18.8 (9.03)	0.036
median (min – max)	14.0 (7 – 39)	17.5 (5 – 39)	
PANSS Negative Items Score			
mean (SD)	23.1 (6.36)	26.2 (6.0)	< 0.001
median (min – max)	25.0 (7 – 34)	27.0 (5 – 38)	
PANSS General Psychopathology Items Score			
mean (SD)	51.7 (11.51)	53.5 (11.01)	0.236
median (min – max)	53.0 (25 – 79)	55.0 (5 – 77)	
PANSS TOTAL Score			
mean (SD)	91.0 (20.34)	98.3 (20.95)	0.008
median (min – max)	91.0 (49 – 149)	98.5 (5 – 149)	
Drop-out patients, N (%)	7 (7.5)	19 (12.7)	-
Analysis Population [1], N (%)	86 (92.5)	131 (87.3)	-

[1] Analysis Population comprising all patients with available BPRS and PANSS scores at both Baseline and End of Study (Month 12) Visits.

ANALYSIS Population N = 217 (completers)

Treatment: BREXPIPRAZOLE (N = 86)

BPRS Total Score Descriptive Analysis

Use of Substances		BPRS Total Score				
		Baseline	Month 1	Month 3	Month 6	Month 12
**NO**	N	38	38	38	38	38
	**Mean**	**62.08**	**34.00**	**30.84**	**29.71**	**26.61**
	SD	13.806	7.874	7.611	6.027	2.881
	Median	60.00	31.00	29.50	28.50	25.50
	Minimum	41	25	24	24	24
	Maximum	105	58	54	45	35
**YES**	N	48	48	48	48	48
	**Mean**	**69.92**	**41.50**	**36.83**	**35.79**	**30.13**
	SD	19.326	17.782	14.164	12.745	6.532
	Median	66.00	33.00	28.50	29.00	27.00
	Minimum	38	24	24	24	23
	Maximum	115	94	73	71	45

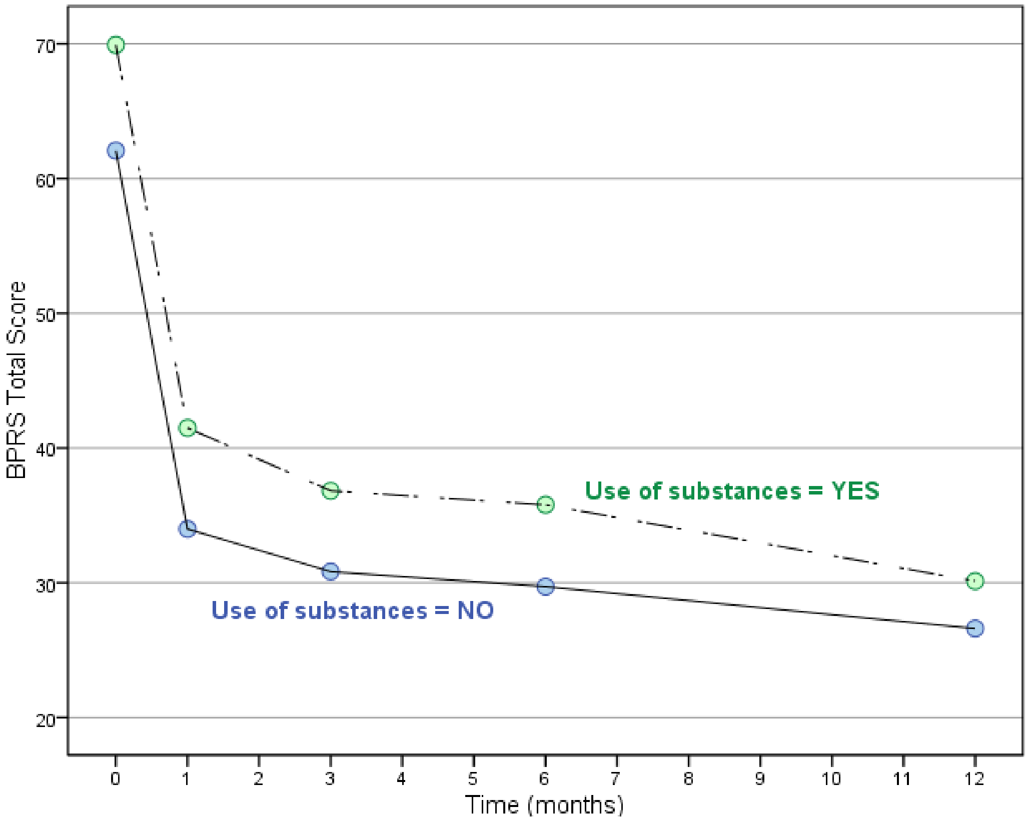

Figure S1. Brexpiprazole group. SUD$^+$ and SUD$^-$ subgroups compared for BPRS total scores.

Treatment: BREXPIPRAZOLE

BPRS Total Score Descriptive Analysis Repeated Measures General Linear Model (GLM) Analysis

Mauchly's Test of Sphericity							
Within Subjects Effect	Mauchly's *W*	Approx. χ^2	df	***p*-value**	*ε*		
					Greenhouse-Geisser	Huynh-Feldt	Lower-bound
Time	.024	300.521	9	**.000**	.440	.465	.250

p-value < 0.05: The assumption of sphericity has been violated, hence the application of the Greenhouse-Geisser correction is needed.

**Tests of Within-Subjects Effects**						
Source	Type III Sum of Squares	df	Mean Square	*F*	***p*-value**	Partial η^2
Time	7381.110	1.759	4195.916	29.943	**.000**	.267
Time × Age	686.611	1.759	390.315	2.785	**.072**	.033
Time × Sex	477.116	1.759	271.224	1.935	**.153**	.023
Time × Use of Substance	6.220	1.759	3.536	.025	**.964**	.000
Error (factor1)	20213.719	144.248	140.132	-	**-**	-

This is the main table corresponding to time analysis:
Time effect was significant (*p*-value < 0.001) on the decrease of total BPRS scores.

Tests of Between-Subjects Effects						
Source	Type III Sum of Squares	df	Mean Square	*F*	***p*-value**	Partial η^2
Intercept	69111.912	1	69111.912	152.660	**.000**	.651
Age	4710.168	1	4710.168	10.404	**.002**	.113
Sex	774.633	1	774.633	1.711	**.195**	.020
Use of Substance	244.943	1	244.943	.541	**.464**	.007
Error	37122.901	82	452.718	-	**-**	-

This table indicates whether the groups differ for the considered parameter independently from time. The two groups differ significantly (*p*-value = 0.002) for age.

Treatment: BREXPIPRAZOLE

PANSS Total Score Descriptive Analysis

Use of Substances		PANSS Total Score				
		Baseline	Month 1	Month 3	Month 6	Month 12
**NO**	N	38	38	38	38	38
	**Mean**	**85.29**	**49.58**	**42.82**	**41.08**	**36.92**
	SD	14.939	15.140	12.105	10.031	6.360
	Median	87.50	48.50	41.00	40.00	37.00
	Minimum	56	32	29	29	28
	Maximum	115	89	72	61	51
**YES**	N	48	48	48	48	48
	**Mean**	**97.65**	**58.15**	**50.50**	**48.46**	**43.04**
	SD	22.230	23.378	17.991	16.627	11.950
	Median	95.50	57.00	47.00	41.00	38.50
	Minimum	49	30	29	29	29
	Maximum	149	124	92	92	72

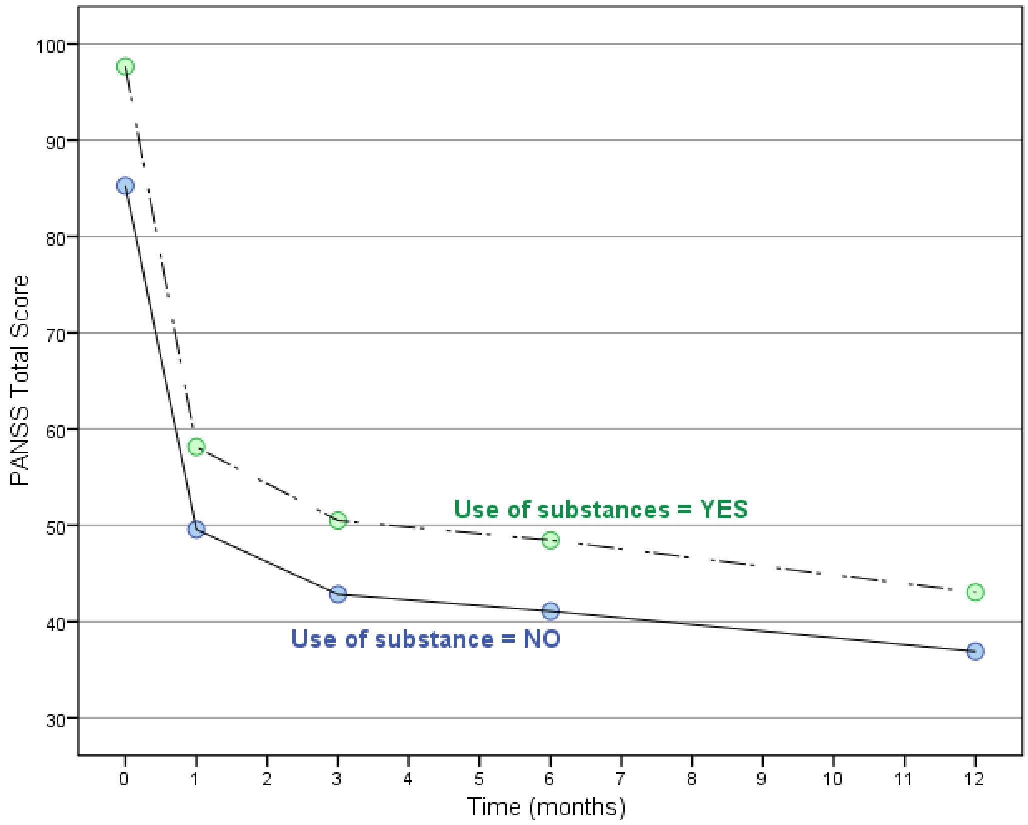

Figure S2. Brexpiprazole group. SUD$^+$ and SUD$^-$ subgroups compared for PANSS total scores.

Treatment: BREXPIPRAZOLE

PANSS Total Score Descriptive Analysis Repeated Measures General Linear Model Analysis

Mauchly's Test of Sphericity							
Within Subjects Effect	Mauchly's *W*	Approx. χ^2	df	***p*-value**	ε		
					Greenhouse-Geisser	Huynh-Feldt	Lower-bound
Time	.038	263.395	9	**.000**	.463	.490	.250

p-value < 0.05: The assumption of sphericity has been violated, hence the application of the Greenhouse-Geisser correction is needed.

Tests of Within-Subjects Effects						
Source	Type III Sum of Squares	df	Mean Square	*F*	***p*-value**	Partial η^2
Time	10379.592	1.851	5606.495	27.157	**.000**	.249
Time × Age	1166.404	1.851	630.028	3.052	**.054**	.036
Time × Sex	272.865	1.851	147.387	.714	**.481**	.009
Time × Use of Substance	556.472	1.851	300.576	1.456	**.237**	.017
Error (factor1)	31341.118	151.811	206.449	-	**-**	-

This is the main table corresponding to time analysis:
Time effect was significant (*p*-value < 0.001) on the decrease of total BPRS scores.

Tests of Between-Subjects Effects						
Source	Type III Sum of Squares	df	Mean Square	*F*	***p*-value**	Partial η^2
Intercept	136209.647	1	136209.647	168.361	**.000**	.672
Age	8445.899	1	8445.899	10.440	**.002**	.113
Sex	2137.099	1	2137.099	2.642	**.108**	.031
Use of Substance	351.051	1	351.051	.434	**.512**	.005
Error	66340.630	82	809.032	-	**-**	-

This table indicates whether the groups differ for the considered parameter independently from time. The two groups differ significantly (*p*-value = 0.002) for age.

Treatment: ARIPIPRAZOLE (N =131)

BPRS Total Score Descriptive Analysis

Use of Substances		BPRS Total Score				
		Baseline	Month 1	Month 3	Month 6	Month 12
**NO**	N	57	57	57	57	57
	**Mean**	**68.05**	**46.58**	**41.46**	**37.68**	**26.02**
	SD	13.197	9.996	10.126	9.947	8.993
	Median	67.00	47.00	41.00	37.00	24.00
	Minimum	42	26	24	24	18
	Maximum	91	70	88	88	60
**YES**	N	74	74	74	74	74
	**Mean**	**73.20**	**54.43**	**51.59**	**50.91**	**42.65**
	SD	20.619	14.899	14.842	16.669	19.464
	Median	75.50	52.50	48.00	47.50	42.00
	Minimum	5	26	26	26	0
	Maximum	115	97	93	93	92

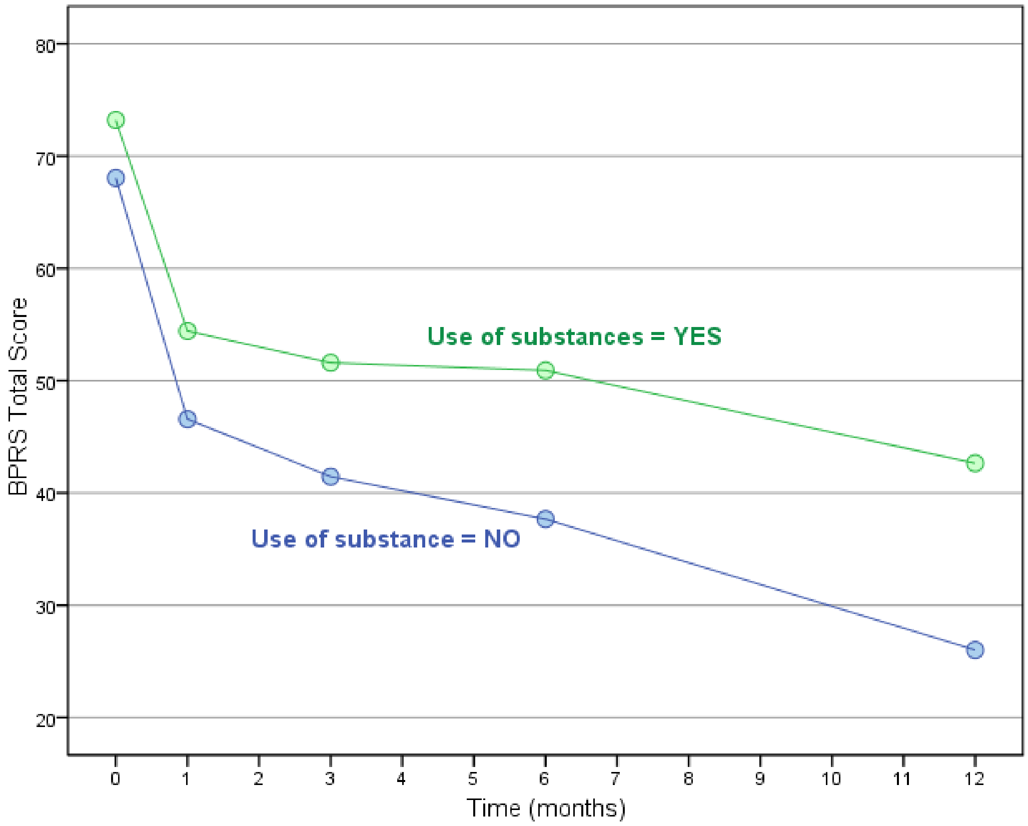

Figure S3. Aripiprazole group. SUD+ and SUD− subgroups compared for BPRS total scores.

Treatment: ARIPIPRAZOLE

BPRS Total Score Descriptive Analysis Repeated Measures General Linear Model (GLM) Analysis

Mauchly's Test of Sphericity							
Within Subjects Effect	Mauchly's W	Approx. χ^2	df	***p*-value**	ε Greenhouse-Geisser	Huynh-Feldt	Lower-bound
Time	.370	124.594	9	.000	.664	.696	.250

p-value < 0.05: The assumption of sphericity has been violated, hence the application of the Greenhouse-Geisser correction is needed.

**Tests of Within-Subjects Effects**						
Source	Type III Sum of Squares	df	Mean Square	*F*	***p*-value**	Partial η^2
Time	2595.541	2.658	976.669	7.516	**.000**	.056
Time × Age	2308.246	2.658	868.563	6.684	**.000**	.050
Time × Sex	565.164	2.658	212.664	1.637	**.186**	.013
Time × Use of Substance	28.222	2.784	10.138	.082	**.963**	.001
Error (factor1)	43854.778	337.508	129.937	-	**-**	-

This is the main table corresponding to time analysis:
Time effect was significant (*p*-value < 0.001) on the decrease of total BPRS scores.
Time×age had a significant effect (*p*-value < 0.001) on the decrease of BPRS Total Scores.

Tests of Between-Subjects Effects						
Source	Type III Sum of Squares	df	Mean Square	*F*	***p*-value**	Partial η^2
Intercept	152851.486	1	152851.486	238.570	**.000**	.653
Age	12042.417	1	12042.417	18.796	**.000**	.129
Sex	1564.858	1	1564.858	2.442	**.121**	.019
Use of Substance	.714	1	.714	.001	**.973**	.000
Error	81368.857	127	640.700	-	-	-

This table indicates whether the groups differ for the considered parameter independently from time. The two groups differ significantly (*p*-value < 0.001) for age.

Treatment: ARIPIPRAZOLE

PANSS Total Score Descriptive Analysis

Use of Substances		PANSS Total Score				
		Baseline	Month 1	Month 3	Month 6	Month 12
**NO**	N	57	57	57	57	57
	**Mean**	**94.44**	**68.95**	**56.39**	**50.81**	**43.61**
	SD	16.252	13.795	12.828	13.552	11.991
	Median	93.00	70.00	57.00	49.00	41.00
	Minimum	64	32	32	30	30
	Maximum	123	107	115	115	88
**YES**	N	74	74	74	74	74
	**Mean**	**99.53**	**75.18**	**70.36**	**68.81**	**62.30**
	SD	24.707	19.049	19.444	21.626	24.997
	Median	104.00	75.00	67.50	64.00	58.00
	Minimum	5	32	32	32	2
	Maximum	149	135	121	121	132

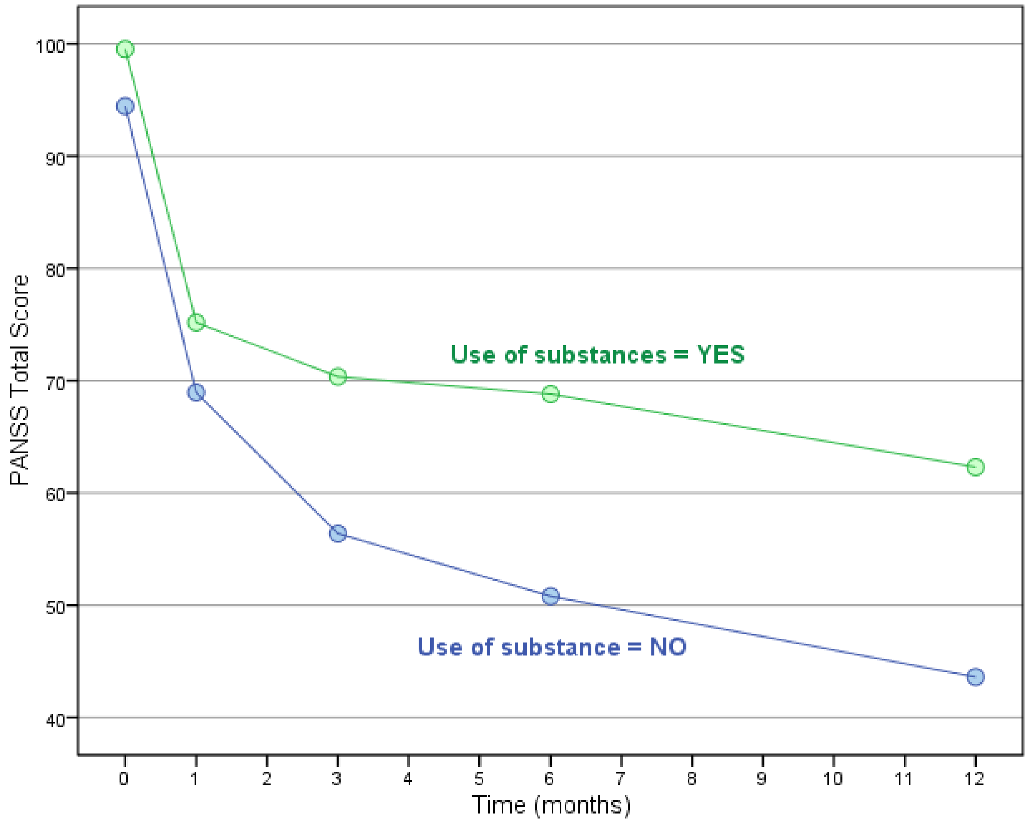

Figure S4. Aripiprazole group. SUD[+] and SUD[–] subgroups compared for PANSS total scores.

Treatment: ARIPIPRAZOLE

PANSS Total Score Descriptive Analysis Repeated Measures General Linear Model Analysis

Mauchly's Test of Sphericity							
Within Subjects Effect	Mauchly's *W*	Approx. χ^2	df	***p*-value**	Epsilon		
					Greenhouse-Geisser	Huynh-Feldt	Lower-bound
Time	.523	81.321	9	**.000**	.751	.789	.250

p-value < 0.05: The assumption of sphericity has been violated, hence the application of the Greenhouse-Geisser correction is needed.

Tests of Within-Subjects Effects						
Source	Type III Sum of Squares	df	Mean Square	*F*	***p*-value**	Partial η^2
Time	6080.906	3.004	2024.126	10.459	**.000**	.076
Time × Age	2351.000	3.004	782.568	4.044	**.008**	.031
Time × Sex	1241.467	3.004	413.242	2.135	**.095**	.017
Time × Use of Substance	431.603	3.004	143.666	.742	**.528**	.006
Error (factor1)	73839.608	381.535	193.533	-	**-**	-

This is the main table corresponding to time analysis:
Time effect was significant (*p*-value < 0.001) on the decrease of total BPRS scores.
Time×age had a significant effect (*p*-value = 0.008) on the decrease of BPRS Total Scores.

Tests of Between-Subjects Effects						
Source	Type III Sum of Squares	df	Mean Square	F	***p*-value**	Partial η^2
Intercept	311655.039	1	311655.039	332.988	**.000**	.724
Age	32066.882	1	32066.882	34.262	**.000**	.212
Sex	1732.138	1	1732.138	1.851	**.176**	.014
Use of Substance	1266.569	1	1266.569	1.353	**.247**	.011
Error	118863.806	127	935.935	-	**-**	-

This table indicates whether the groups differ for the considered parameter independently from time. The two groups differ significantly (*p*-value < 0.001) for age.

BPRS Total Score Descriptive Analysis

BREXPIPRAZOLE					
Use of Substances		Baseline	Month 12	Change from Baseline	Change from Baseline (%)
No	N	38	38	38	38
	Mean	62.08	26.61	-35.47	-55.19
	SD	13.806	2.881	13.590	11.014
	Median	60.00	25.50	-35.00	-58.33
	Minimum	41	24	-76	-72
	Maximum	105	35	-9	-22
Yes	N	48	48	48	48
	Mean	69.92	30.13	-39.79	-54.76
	SD	19.326	6.532	17.425	12.430
	Median	66.00	27.00	-38.00	-55.74
	Minimum	38	23	-82	-76
	Maximum	115	45	0	0
Total	N	86	86	86	86
	Mean	66.45	28.57	-37.88	-54.95
	SD	17.459	5.504	15.904	11.760
	Median	64.00	26.00	-36.00	-56.77
	Minimum	38	23	-82	-76
	Maximum	115	45	0	0

ARIPIPRAZOLE					
Use of Substances		Baseline	Month 12	Change from Baseline	Change from Baseline (%)
No	N	57	57	57	57
	Mean	68.05	26.02	-42.04	-60.99
	SD	13.197	8.993	13.774	13.432
	Median	67.00	24.00	-41.00	-66.10
	Minimum	42	18	-68	-75
	Maximum	91	60	-5	-11
Yes	N	74	74	74	74

	Mean	73.20	42.65	-30.55	-41.31
	SD	20.619	19.464	21.746	23.975
	Median	75.50	42.00	-30.00	-44.45
	Minimum	5	0	-87	-100
	Maximum	115	92	-1	-2
Total	N	131	131	131	131
	Mean	70.96	35.41	-35.55	-49.87
	SD	17.897	17.779	19.491	22.280
	Median	69.00	26.00	-37.00	-58.33
	Minimum	5	0	-87	-100
	Maximum	115	92	-1	-2

PANSS Total Score Descriptive Analysis

BREXPIPRAZOLE					
Use of Substances		Baseline	Month 12	Change from Baseline	Change from Baseline (%)
No	N	38	38	38	38
	Mean	85.29	36.92	-48.37	-55.71
	SD	14.939	6.360	14.406	9.096
	Median	87.50	37.00	-48.00	-53.52
	Minimum	56	28	-77	-68
	Maximum	115	51	-25	-40
Yes	N	48	48	48	48
	Mean	97.65	43.04	-54.60	-54.54
	SD	22.230	11.950	20.583	13.417
	Median	95.50	38.50	-55.00	-56.31
	Minimum	49	29	-101	-74
	Maximum	149	72	0	0
Total	N	86	86	86	86
	Mean	92.19	40.34	-51.85	-55.06
	SD	20.212	10.292	18.284	11.658
	Median	91.50	38.00	-52.50	-55.91
	Minimum	49	28	-101	-74
	Maximum	149	72	0	0

ARIPIPRAZOLE					
Use of Substances		Baseline	Month 12	Change from Baseline	Change from Baseline (%)
No	N	57	57	57	57
	Mean	94.44	43.61	-50.82	-53.31
	SD	16.252	11.991	15.893	11.768
	Median	93.00	41.00	-50.00	-55.71
	Minimum	64	30	-78	-67
	Maximum	123	88	-3	-4
Yes	N	74	74	74	74
	Mean	99.53	62.30	-37.23	-37.06
	SD	24.707	24.997	26.155	20.609
	Median	104.00	58.00	-35.00	-39.44
	Minimum	5	2	-111	-74
	Maximum	149	132	1	1
Total	N	131	131	131	131
	Mean	97.31	54.17	-43.15	-44.13
	SD	21.517	22.345	23.210	19.069
	Median	98.00	44.00	-44.00	-50.00

	Minimum	5	2	-111	-74
	Maximum	149	132	1	1

PANSS Positive Items Score Descriptive Analysis

BREXPIPRAZOLE					
Use of Substances		Baseline	Month 12	Change from Baseline	Change from Baseline (%)
No	N	38	38	38	38
	Mean	12.95	7.42	-5.53	-34.12
	SD	5.594	.858	5.017	22.297
	Median	10.50	7.00	-3.50	-33.18
	Minimum	7	7	-18	-72
	Maximum	25	10	0	0
Yes	N	48	48	48	48
	Mean	19.35	8.48	-10.88	-45.61
	SD	10.003	2.212	9.169	24.178
	Median	17.50	7.00	-7.00	-46.74
	Minimum	7	6	-30	-79
	Maximum	39	15	0	0
Total	N	86	86	86	86
	Mean	16.52	8.01	-8.51	-40.53
	SD	8.899	1.818	8.036	23.928
	Median	14.50	7.00	-7.00	-43.61
	Minimum	7	6	-30	-79
	Maximum	39	15	0	0

ARIPIPRAZOLE					
Use of Substances		Baseline	Month 12	Change from Baseline	Change from Baseline (%)
No	N	57	57	57	57
	Mean	14.49	8.42	-6.07	-35.66
	SD	6.574	2.699	5.267	18.059
	Median	13.00	8.00	-4.00	-30.77
	Minimum	7	6	-21	-74
	Maximum	30	20	-1	-6
Yes	N	74	74	74	74
	Mean	21.61	12.84	-8.77	-36.30
	SD	9.536	6.573	7.961	22.161
	Median	20.50	12.00	-7.00	-39.23
	Minimum	5	4	-30	-78
	Maximum	39	33	1	7
Total	N	131	131	131	131
	Mean	18.51	10.92	-7.60	-36.02
	SD	9.068	5.677	7.025	20.405
	Median	17.00	9.00	-7.00	-36.84
	Minimum	5	4	-30	-78
	Maximum	39	33	1	7

PANSS Negative Items Score Descriptive Analysis

BREXPIPRAZOLE					
Use of Substances		Baseline	Month 12	Change from Baseline	Change from Baseline (%)
No	N	38	38	38	38
	Mean	22.00	8.66	-13.34	-55.91
	SD	6.858	2.317	5.997	19.806

	Median	25.00	7.50	-15.00	-61.03
	Minimum	7	7	-21	-74
	Maximum	34	15	0	0
Yes	N	48	48	48	48
	Mean	24.15	10.19	-13.96	-55.93
	SD	5.728	3.541	5.323	16.764
	Median	25.00	9.00	-15.00	-60.74
	Minimum	7	7	-22	-74
	Maximum	33	19	0	0
Total	N	86	86	86	86
	Mean	23.20	9.51	-13.69	-55.92
	SD	6.306	3.139	5.605	18.059
	Median	25.00	8.00	-15.00	-60.74
	Minimum	7	7	-22	-74
	Maximum	34	19	0	0

ARIPIPRAZOLE					
Use of Substances		Baseline	Month 12	Change from Baseline	Change from Baseline (%)
No	N	57	57	57	57
	Mean	26.00	11.72	-14.28	-54.62
	SD	4.648	3.853	4.419	12.769
	Median	26.00	11.00	-14.00	-56.52
	Minimum	16	7	-23	-75
	Maximum	36	26	-1	-5
Yes	N	74	74	74	74
	Mean	25.78	15.74	-10.04	-38.69
	SD	7.185	7.349	6.369	20.618
	Median	27.00	14.00	-10.00	-40.97
	Minimum	5	4	-24	-73
	Maximum	38	34	-1	-3
Total	N	131	131	131	131
	Mean	25.88	13.99	-11.89	-45.62
	SD	6.189	6.382	5.970	19.282
	Median	26.00	12.00	-12.00	-51.61
	Minimum	5	4	-24	-75
	Maximum	38	34	-1	-3

PANSS General Psychopathology Items Score **Descriptive Analysis**

BREXPIPRAZOLE					
Use of Substances		Baseline	Month 12	Change from Baseline	Change from Baseline (%)
No	N	38	38	38	38
	Mean	50.39	20.84	-29.55	-57.43
	SD	10.681	5.059	10.091	10.541
	Median	53.00	21.00	-29.50	-55.22
	Minimum	25	12	-50	-76
	Maximum	67	31	-13	-34
Yes	N	48	48	48	48
	Mean	54.23	24.37	-29.85	-53.91
	SD	11.293	7.775	11.385	14.814
	Median	53.50	22.00	-28.50	-53.82
	Minimum	31	15	-54	-77
	Maximum	79	49	0	0

Total	N	86	86	86	86
	Mean	52.53	22.81	-29.72	-55.47
	SD	11.129	6.905	10.771	13.146
	Median	53.00	21.00	-29.00	-54.42
	Minimum	25	12	-54	-77
	Maximum	79	49	0	0

ARIPIPRAZOLE					
Use of Substances		Baseline	Month 12	Change from Baseline	Change from Baseline (%)
No	N	57	57	57	57
	Mean	53.95	25.74	-28.21	-51.04
	SD	10.436	5.884	10.266	12.653
	Median	56.00	25.00	-30.00	-54.29
	Minimum	29	16	-42	-66
	Maximum	68	48	-1	-3
Yes	N	74	74	74	74
	Mean	52.31	33.81	-18.50	-34.49
	SD	12.151	12.840	13.673	20.853
	Median	53.50	33.00	-17.00	-36.56
	Minimum	5	4	-57	-74
	Maximum	77	65	1	2
Total	N	131	131	131	131
	Mean	53.02	30.30	-22.73	-41.69
	SD	11.423	11.119	13.180	19.519
	Median	55.00	26.00	-22.00	-46.67
	Minimum	5	4	-57	-74
	Maximum	77	65	1	2

COMPARISON: Brexpiprazole vs. Aripiprazole (ANCOVA Analysis)

- **BPRS Total Score**

Tests of Between-Subjects Effects						
Dependent Variable: BPRS Total Score Month 12 change from Baseline						
Source	Type III Sum of Squares	df	Mean Square	*F*	***p*-value**	Partial η^2
Corrected Model	33522.830	2	16761.415	95.278	**.000**	.471
Intercept	1764.439	1	1764.439	10.030	**.002**	.045
Baseline BPRS	33239.984	1	33239.984	188.947	**.000**	.469
**Treatment**	**1544.441**	**1**	**1544.441**	**8.779**	**.003**	**.039**
Error	37647.280	214	175.922	-	-	-
Total	359867.000	217	-	-	-	-
Corrected Total	71170.111	216	-	-	-	-

R^2 = .471 (Adjusted R^2 = .466)

Estimates Marginal Means (EMMs)				
Dependent Variable: BPRS Total Score Month 12 change from Baseline				
Treatment	Mean	SEM	95% C.I.	
			Lower	Upper
Brexpiprazole	-39.793	1.437	-42.625	-36.960
Aripiprazole	-34.296	1.162	-36.588	-32.005

Pairwise Comparisons					
Dependent Variable: BPRS Total Score Month 12 change from Baseline					
	Mean Difference	SEM	***p*-value**	95% C.I. for Difference	
				Lower	Upper
Brexpiprazole - Aripiprazole	-5.497	1.855	**.003**	-9.153	-1.840

Based on estimated marginal means
The mean difference is significant at the 0.05 level.
Adjustment for multiple comparisons: Least Significant Difference (equivalent to no adjustments).

BPRS Total Scores: A significantly greater decrease is observed in the group of patients who received Brexpiprazole compared to patients treated with Aripiprazole (*p*-value = 0.003; Brexpiprazole -39.8 vs. Aripiprazole -34.3).

COMPARISON: Brexpiprazole vs. Aripiprazole (ANCOVA Analysis)

- **PANSS Total Score**

Tests of Between-Subjects Effects						
Dependent Variable: PANSS Total Score Month 12 change from Baseline						
Source	Type III Sum of Squares	df	Mean Square	*F*	***p*-value**	Partial η^2
Corrected Model	55105.844	3	18368.615	82.762	**.000**	.538
Intercept	10665.014	1	10665.014	48.052	**.000**	.184
Baseline PANSS	47626.541	1	47626.541	214.586	**.000**	.502
**Treatment**	**13590.642**	**1**	**13590.642**	**61.234**	**.000**	**.223**
Error	7902.463	1	7902.463	35.605	**.000**	.143
Total	47274.469	213	221.946	-	-	-
Corrected Total	573497.000	217		-	-	-

R^2 = .538 (Adjusted R^2 = .532)

Estimates Marginal Means (EMMs)				
Dependent Variable: PANSS Total Score Month 12 change from Baseline				
Treatment	Mean	SEM	95% C.I.	
			Lower	Upper
Brexpiprazole	-54.099	1.614	-57.281	-50.918
Aripiprazole	-41.668	1.306	-44.241	-39.094

Pairwise Comparisons					
Dependent Variable: PANSS Total Score Month 12 change from Baseline					
	Mean Difference	SEM	*p*-value	95% C.I. for Difference	
				Lower	Upper
Brexpiprazole - Aripiprazole	-12.432	2.083	**.000**	-16.538	-8.325

Based on estimated marginal means
The mean difference is significant at the .05 level.
Adjustment for multiple comparisons: Least Significant Difference (equivalent to no adjustments).

PANSS Total Scores: A significantly greater decrease is observed in the group of patients treated with Brexpiprazole compared to the group of patients treated with Aripiprazole (*p*-value < 0.001; Brexpiprazole -54.1 vs. Aripiprazole -41.7).

COMPARISON: **Brexpiprazole vs. Aripiprazole (ANCOVA Analysis)**
Use of substances (YES vs. NO)

- **BPRS Total Score**

Tests of Between-Subjects Effects						
Dependent Variable: BPRS Total Score Month 12 change from Baseline						
Source	Type III Sum of Squares	df	Mean Square	*F*	***p*-value**	Partial η^2
Corrected Model	40999.238	4	10249.810	72.022	**.000**	.576
Intercept	2488.947	1	2488.947	17.489	**.000**	.076
Baseline BPRS	36076.732	1	36076.732	253.498	**.000**	.545
Use of substances	3497.765	1	3497.765	24.578	**.000**	.104
**Treatment**	**1147.892**	**1**	**1147.892**	**8.066**	**.005**	**.037**
Trt × Substance use	2432.625	1	2432.625	17.093	**.000**	.075
Error	30170.872	212	142.315	-	-	-
Total	359867.000	217	-	-	-	-
Corrected Total	71170.111	216	-	-	-	-

R^2 = 0.576 (Adjusted R^2 = 0.568)

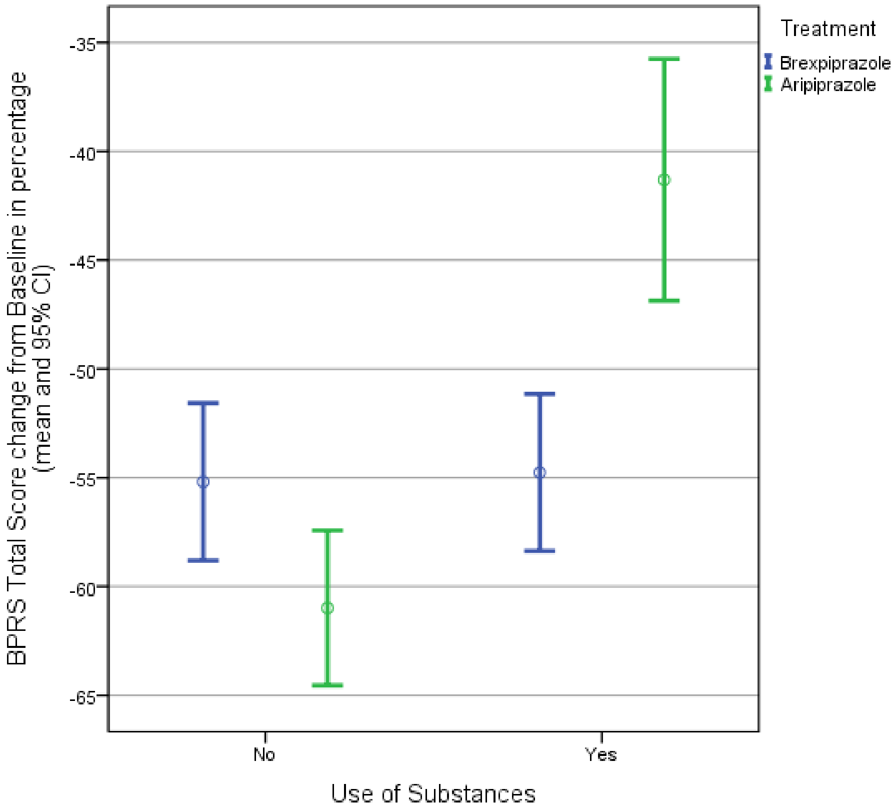

Figure S5. SCZ-SUD⁻ and SUD⁺ Brexpiprazole and Aripiprazole groups compared for % changes from baseline in BPRS total scores.

COMPARISON: Brexpiprazole vs. Aripiprazole (ANCOVA Analysis)
Use of substances (YES vs. NO)

- **PANSS Total Score**

Tests of Between-Subjects Effects						
Dependent Variable: PANSS Total Score Month 12 change from Baseline						
Source	Type III Sum of Squares	df	Mean Square	*F*	***p*-value**	Partial η^2
Corrected Model	50759.364	4	12689.841	52.115	**.000**	.496
Intercept	2137.078	1	2137.078	8.777	**.003**	.040
Baseline PANSS Tot	40050.713	1	40050.713	164.483	**.000**	.437
Use of substances	4399.647	1	4399.647	18.069	**.000**	.079
**Treatment**	**6219.419**	**1**	**6219.419**	**25.542**	**.000**	**.108**
Trt × Substance use	2857.523	1	2857.523	11.735	**.001**	.052
Error	51620.949	212	243.495	-	-	-
Total	573497.000	217	-	-	-	-
Corrected Total	102380.313	216	-	-	-	-

R^2 = .496 (Adjusted R^2 = 0.486)

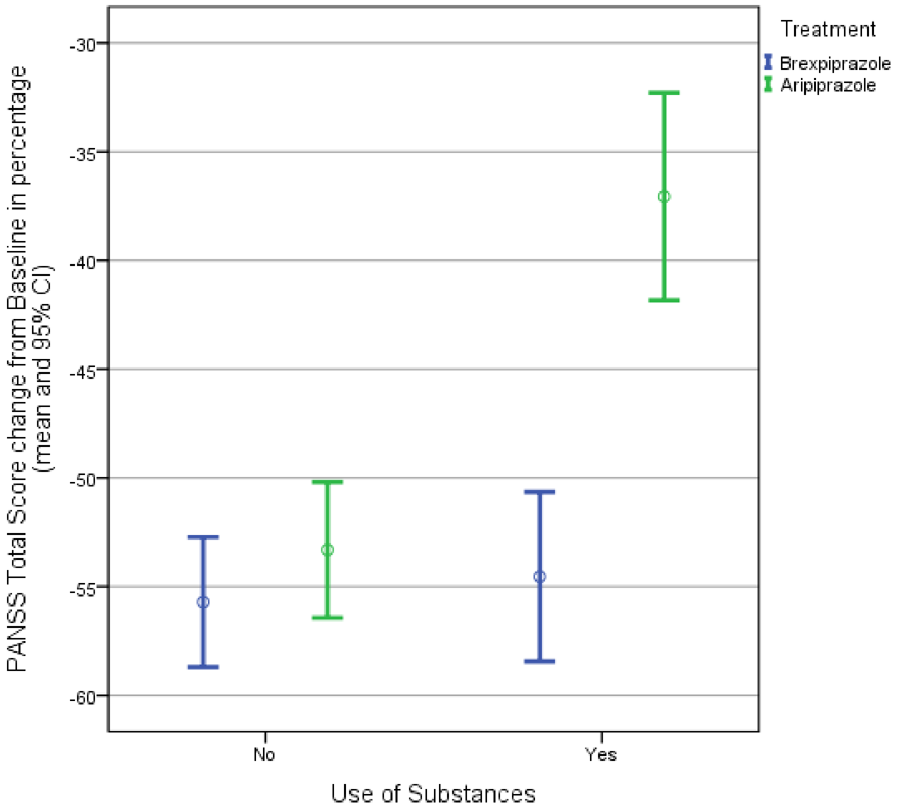

Figure S6. SCZ-SUD⁻ and SUD⁺ Brexpiprazole and Aripiprazole groups compared for % changes from baseline in PANSS total scores.

COMPARISON: Brexpiprazole *vs.* Aripiprazole (ANCOVA Analysis)
Use of substances (YES *vs.* NO)

- **PANSS Positive Items**

**Tests of Between-Subjects Effects**						
Dependent Variable: PANSS Positive Items Month 12 change from Baseline						
Source	Type III Sum of Squares	df	Mean Square	*F*	***p*-value**	Partial η^2
Corrected Model	9116.390	4	2279.098	170.596	**.000**	.763
Intercept	977.053	1	977.053	73.135	**.000**	.256
Baseline PANSS Pos	8231.301	1	8231.301	616.134	**.000**	.744
Use of substances	44.062	1	44.062	3.298	.071	.015
**Treatment**	**242.514**	**1**	**242.514**	**18.153**	**.000**	**.079**
Trt × Substance use	128.926	1	128.926	9.650	**.002**	.044
Error	2832.236	212	13.360	-	-	-
Total	25693.000	217	-	-	-	-
Corrected Total	11948.627	216	-	-	-	-

R^2 = .763 (Adjusted R^2 = 0.758)

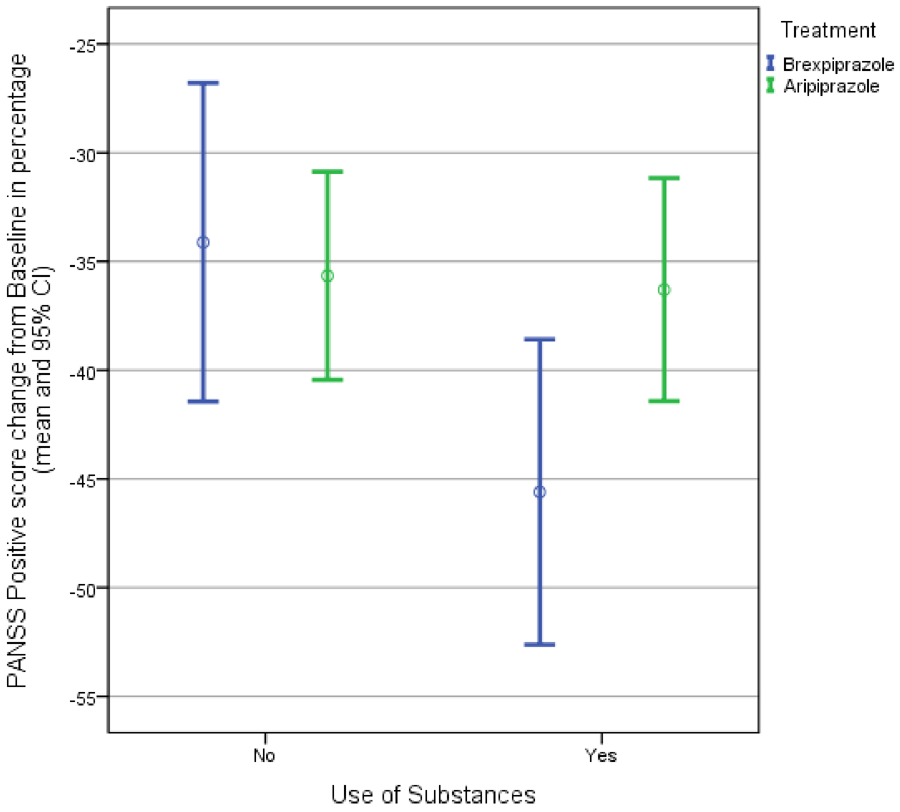

Figure S7. SCZ-SUD⁻ and SUD⁺ Brexpiprazole and Aripiprazole groups compared for % changes from baseline in PANSS-Positive scores.

COMPARISON: Brexpiprazole vs. Aripiprazole (ANCOVA Analysis)
Use of substances (YES vs. NO)

- **PANSS Negative Items**

Tests of Between-Subjects Effects						
Dependent Variable: PANSS Negative Items Month 12 change from Baseline						
Source	Type III Sum of Squares	df	Mean Square	*F*	***p*-value**	Partial η^2
Corrected Model	3438.473[a]	4	859.618	45.180	**.000**	.460
Intercept	14.178	1	14.178	.745	.389	.004
Baseline PANSS Neg	2683.209	1	2683.209	141.024	**.000**	.399
Use of substances	283.676	1	283.676	14.909	**.000**	.066
**Treatment**	**467.329**	**1**	**467.329**	**24.562**	**.000**	**.104**
Trt × Substance use	156.200	1	156.200	8.210	**.005**	.037
Error	4033.647	212	19.027	-	-	-
Total	41918.000	217	-	-	-	-
Corrected Total	7472.120	216	-	-	-	-

R^2 = 0.460 (Adjusted R^2 = 0.450)

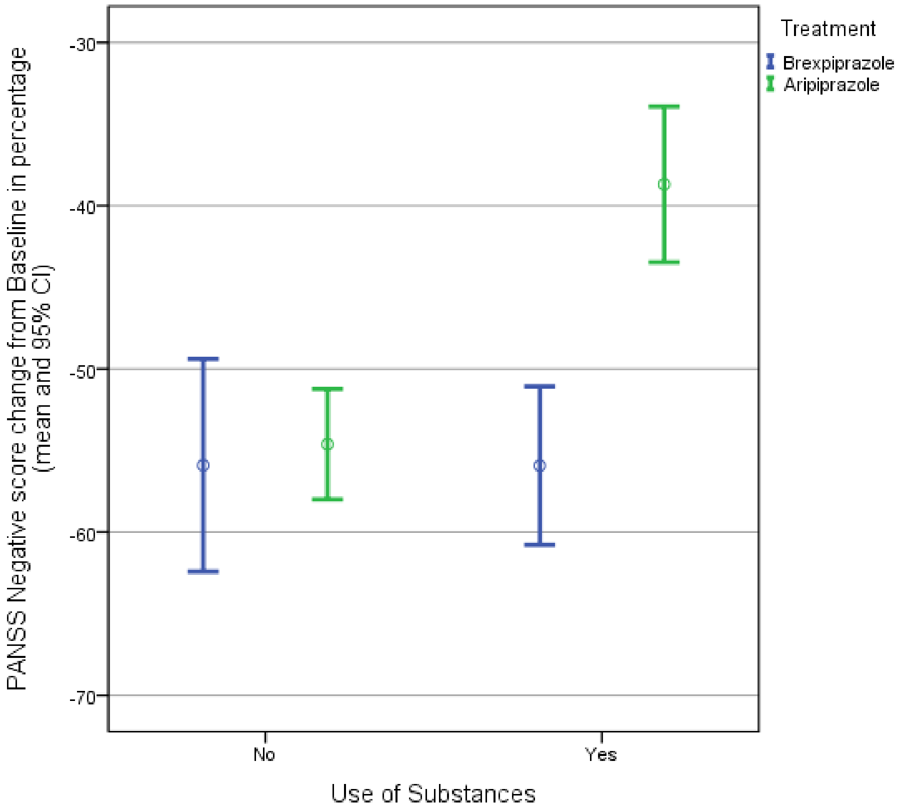

Figure 8. SCZ-SUD⁻ and SUD⁺ Brexpiprazole and Aripiprazole groups compared for % changes from baseline in PANSS-Negative scores.

COMPARISON: Brexpiprazole *vs.* Aripiprazole (ANCOVA Analysis)
Use of substances (YES vs. NO)

- **PANSS General Psychopathology Items**

**Tests of Between-Subjects Effects**						
Dependent Variable: PANSS General Psychopathology Items Month 12 change from Baseline						
Source	Type III Sum of Squares	df	Mean Square	*F*	***p*-value**	Partial η^2
Corrected Model	19425.543[a]	4	4856.386	66.164	**.000**	.555
Intercept	1158.976	1	1158.976	15.790	**.000**	.069
Baseline PANSS Gen	13846.642	1	13846.642	188.647	**.000**	.471
Use of substances	1538.133	1	1538.133	20.956	**.000**	.090
**Treatment**	**2454.859**	**1**	**2454.859**	**33.445**	**.000**	**.136**
Trt × Substance use	469.289	1	469.289	6.394	**.012**	.029
Error	15560.705	212	73.400	-	-	-
Total	176065.000	217	-	-	-	-
Corrected Total	34986.249	216	-	-	-	-

R^2 = .555 (Adjusted R^2 = .547)

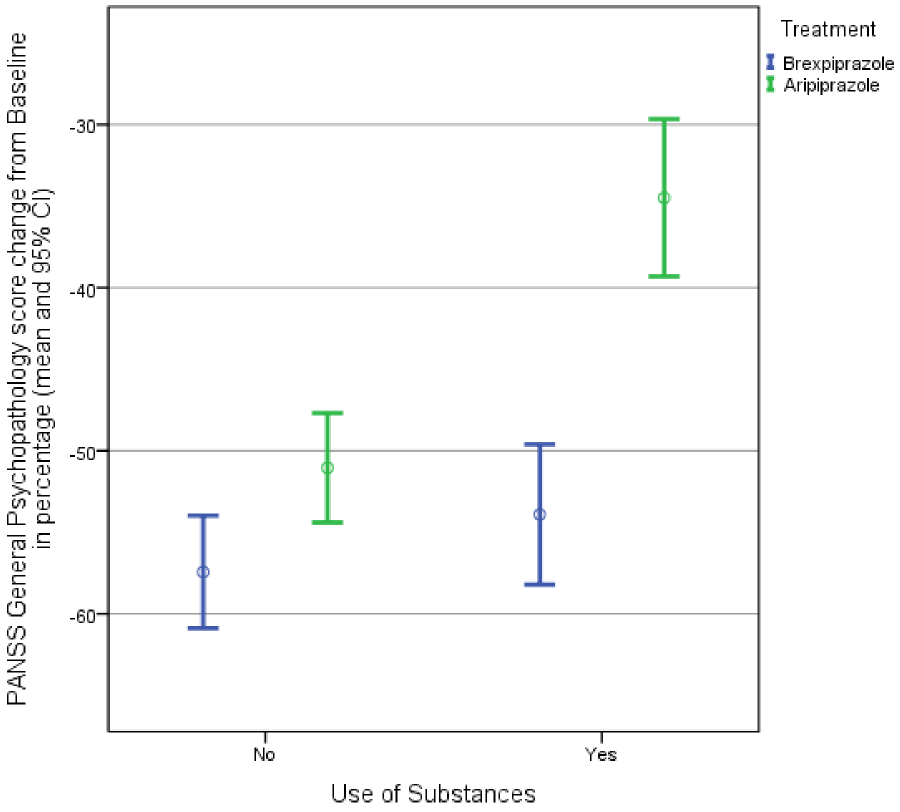

Figure S9. SCZ-SUD⁻ and SUD⁺ Brexpiprazole and Aripiprazole groups compared for % changes from baseline in PANSS-General Psychopathology scores.

Statistical analysis

Descriptive statistics were first calculated, with categorical variables expressed as count and percentage, and continuous variables reported as mean, standard deviation, median, minimum and maximum. To assess the sample distribution, normality was checked. Skewness and kurtosis values were found to be within the acceptable range confirming a normal distribution. Demographic and baseline clinical characteristics were compared between the two study groups using analysis of variance (ANOVA) for continuous variables and χ^2 test for categorical variables. If demographic and baseline clinical characteristics differed between two groups, analysis of covariance (ANCOVA) was conducted using these characteristics as covariates. Analysis of covariance (ANCOVA) was used to compare clinical outcomes (BPRS Total Score, PANSS Total Score, PANSS Positive Scale, PANSS Negative Scale, and PANSS General Psychopathology Scale) change from baseline including the treatment group and the use of substances as factors and baseline scale value as covariate. The significance level was set at p = 0.05, and Bonferroni correction was performed when testing for multiple dependent variables. All tests were two-sided. For all statistical analyses the Statistical Package for Social Science (SPSS) version 29.0 (IBM, Armonk, NY, USA) was used.

STROBE (STrengthening the Reporting of Observational studies in Epidemiology) Statement—checklist of items that should be included in reports of observational studies

	Item No.	Recommendation	Page No.	Relevant text from manuscript
**Title and abstract**	1	(*a*) Indicate the study's design with a commonly used term in the title or the abstract	1	A 12-month Real-World Naturalistic Study
		(*b*) Provide in the abstract an informative and balanced summary of what was done and what was found	1	Brexpiprazole and aripiprazole were associated with psychotic symptom and global psychopathology improvement over 12 months; Among patients with comorbid SUD, brexpiprazole showed greater improvement on BPRS and PANSS outcomes than aripiprazole
**Introduction**				
Background/ rationale	2	Explain the scientific background and rationale for the investigation being reported	2	Since dopaminergic dysregulation also underlies craving and reward-seeking behaviours, partial D_2 agonists may offer therapeutic advantages in this subgroup
Objectives	3	State specific objectives, including any prespecified hypotheses	2	Naturalistic study to compare the longitudinal real-world effectiveness of brexpiprazole *vs*. aripiprazole in patients with SCZ, with or without comorbid SUD over 12 months
**Methods**				
Study design	4	Present key elements of study design early in the paper	2	Observational study on 243 hospitalised patients with SCZ diagnosis
Setting	5	Describe the setting, locations, and relevant dates, including periods of recruitment, exposure, follow-up, and data collection	2	Recruitment January 2022-October 2022; Villa Von Siebenthal neuropsychiatric hospital; 12-month follow-up
Participants	6	(*a*) *Cohort study*—Give the eligibility criteria, and the sources and methods of selection of participants. Describe methods of follow-up<br><br>*Case-control study*—Give the eligibility criteria, and the sources and methods of case ascertainment and control selection. Give the rationale for the choice of cases and controls<br><br>*Cross-sectional study*—Give the eligibility criteria, and the sources and methods of selection of participants	2-3	Hospitalised patients; Included patients were adults and ranged from 18 to 78 years-old and had a DSM-5/DSM-5-TR diagnosis of SCZ, with or without SUD
		(*b*) *Cohort study*—For matched studies, give matching criteria and number of exposed and unexposed<br><br>*Case-control study*—For matched studies, give matching criteria and the number of controls per case		
Variables	7	Clearly define all outcomes, exposures, predictors, potential confounders, and effect modifiers. Give diagnostic criteria, if applicable	3-4	DSM-5/-5-TR; Oral brexpiprazole or aripiprazole; SUD, age
Data sources-measurement	8×	For each variable of interest, give sources of data and details of methods of assessment (measurement). Describe comparability of assessment methods if there is more than one group	3-4	PANSS, BPRS

Bias	9	Describe any efforts to address potential sources of bias		N/A
Study size	10	Explain how the study size was arrived at		Inclusion of eligible
Quantitative variables	11	Explain how quantitative variables were handled in the analyses. If applicable, describe which groupings were chosen and why	3-4	PANSS and BPRS; SUD-positive and SUD-negaive
Statistical methods	12	(*a*) Describe all statistical methods, including those used to control for confounding	4	If demographic and baseline clinical characteristics differed between two groups, analysis of covariance (ANCOVA) was conducted using these characteristics as covariates. Analy-sis of covariance (ANCOVA) was used to compare clinical outcomes (BPRS Total Score, PANSS Total Score, PANSS Positive Scale, PANSS Negative Scale, and PANSS General Psychopathology Scale) change from baseline including the treatment group and the use of substances as factors and baseline scale value as covariate
		(*b*) Describe any methods used to examine subgroups and interactions		
		(*c*) Explain how missing data were addressed		
		(*d*) *Cohort study*—If applicable, explain how loss to follow-up was addressed<br>*Case-control study*—If applicable, explain how matching of cases and controls was addressed<br>*Cross-sectional study*—If applicable, describe analytical methods taking account of sampling strategy		
		(*e*) Describe any sensitivity analyses		N/A
**Results**				
Participants	13×	(a) Report numbers of individuals at each stage of study—eg numbers potentially eligible, examined for eligibility, confirmed eligible, included in the study, completing follow-up, and analysed	4	243 patients with SCZ, 93 (38.3%) on brexipiprazole and 150 (61.7%) on aripiprazole; 217 (86 in the brexipiprazole group; 131 in the aripiprazole group) were analysed because 26 (10.7%) patients dropped out from the study
		(b) Give reasons for non-participation at each stage	3	26 dropped out (10.7%). Of them, 7 were treated with brexpiprazole (3 withdrew at their own request due to reported subjective agitation and 4 were lost to follow-up) and 19 with aripiprazole (13 withdrew at their own request due to reported subjective agitation and 6 were lost at follow-up).
		(c) Consider use of a flow diagram		N/A
Descriptive data	14×	(a) Give characteristics of study participants (eg demographic, clinical, social) and information on exposures and potential confounders	5	Table 1
		(b) Indicate number of participants with missing data for each variable of interest	4-5	Table 1 and text above
		(c) *Cohort study*—Summarise follow-up time (eg, average and total amount)		
Outcome data	15×	*Cohort study*—Report numbers of outcome events or summary measures over time		
		Case-control study—Report numbers in each exposure category, or summary measures of exposure		
		Cross-sectional study—Report numbers of outcome events or summary measures		
Main results	16	(*a*) Give unadjusted estimates and, if applicable, confounder-adjusted estimates and their precision (eg, 95% confidence interval). Make clear which confounders were adjusted for and why they were included	4-10	Supplement; Age and SUD, pp. S5 and S7; 95%C.I. S11 and S12
		(*b*) Report category boundaries when continuous variables were categorized		N/A
		(*c*) If relevant, consider translating estimates of relative risk into absolute risk for a meaningful time period		N/A
Other analyses	17	Report other analyses done—eg analyses of subgroups and interactions, and sensitivity analyses	4-10,	Interaction of Time × SUD

			Suppl	
**Discussion**				
Key results	18	Summarise key results with reference to study objectives	10	12-month naturalistic study comparing brexpiprazole and aripiprazole in patients with SCZ with or without comorbid SUD. Both medications effective through 12 months for positive and negative symptoms and global psychopathology. When stratifying by substance use, with brexpiprazole and aripiprazole not different in effectiveness in patients without SUD, and brexpiprazole superior to aripiprazole in patients with SUD. This was adjusted for the baseline values of the scales.
Limitations	19	Discuss limitations of the study, taking into account sources of potential bias or imprecision. Discuss both direction and magnitude of any potential bias	13	Naturalistic design, confounders like concomitant medications unaccounted for. Single-centre study, results may not generalise to other populations, needing replication. Lack of comparison with cariprazine, no use of placebo. No outcome stratification by gender, duration of illness, or treatment history (drug-naïve vs. switched patients). No differentiation for different substances in the SUD population
Interpretation	20	Give a cautious overall interpretation of results considering objectives, limitations, multiplicity of analyses, results from similar studies, and other relevant evidence	13-14	Single-centre; both drugs fit for SCZ, brexpiprazole better for SCZ with SUD
Generalisability	21	Discuss the generalisability (external validity) of the study results	13	Single-centre
**Other information**				
Funding	22	Give the source of funding and the role of the funders for the present study and, if applicable, for the original study on which the present article is based	14	N/A, no funding

×Give information separately for cases and controls in case-control studies and, if applicable, for exposed and unexposed groups in cohort and cross-sectional studies.

Note: An Explanation and Elaboration article discusses each checklist item and gives methodological background and published examples of transparent reporting. The STROBE checklist is best used in conjunction with this article (freely available on the Web sites of PLoS Medicine at http://www.plosmedicine.org/, Annals of Internal Medicine at http://www.annals.org/, and Epidemiology at http://www.epidem.com/). Information on the STROBE Initiative is available at www.strobe-statement.org.

Literature for the STROBE Statement

Aghazadeh-Attari, J.; Mobaraki, K.; Ahmadzadeh, J.; Mansorian, B.; Mohebbi, I. Quality of observational studies in prestigious journals of occupational medicine and health based on Strengthening the Reporting of Observational Studies in Epidemiology (STROBE) Statement: a cross-sectional study. *BMC Res. Notes* **2018**, *11*(1), 266. doi: 10.1186/s13104-018-3367-9.

Bastuji-Garin, S.; Sbidian, E.; Gaudy-Marqueste, C.; Ferrat, E.; Roujeau, J.-C.; Richard, M.-A.; Canoui-Poitrine, F.; European Dermatology Network (EDEN). Impact of STROBE statement publication on quality of observational study reporting: interrupted time series versus before-after analysis. *PLoS One* **2013**, *8*(8), e64733. doi: 10.1371/journal.pone.0064733.

Gallo, V.; Egger, M.; McCormack, V.; Farmer, P.B.; Ioannidis, J.P.; Kirsch-Volders, M.; Matullo, G.; Phillips, D.H.; Schoket, B.; Stromberg, U.; Vermeulen, R.; Wild, C.; Porta, M.; Vineis, P.; STROBE Statement. STrengthening the Reporting of OBservational studies in Epidemiology--Molecular Epidemiology (STROBE-ME): an extension of the STROBE Statement. *PLoS Med.* **2011**, *8*(10), e1001117. doi: 10.1371/journal.pmed.1001117.

Hawwash, D.; Sharp, M.K.; Argaw, A.; Kolsteren, P.; Lachat, C. Usefulness of applying research reporting guidelines as Writing Aid software: a crossover randomised controlled trial. *BMJ Open* **2019**, *9*(11), e030943. doi: 10.1136/bmjopen-2019-030943.

Hörnell, A.; Berg, C.; Forsum, E.; Larsson, C.; Sonestedt, E.; Åkesson, A.; Lachat, C.; Hawwash, D.; Kolsteren, P.; Byrnes, G.; De Keyzer, W.; Van Camp, J.; Cade, J.E.; Greenwood, D.C.; Slimani, N.; Cevallos, M.; Egger, M.; Huybrechts, I.; Wirfält, E. Perspective: An extension of the STROBE Statement for Observational Studies in Nutritional Epidemiology (STROBE-nut): Explanation and elaboration. *Adv. Nutr.* **2017**, *8*(5), 652–678. doi: 10.3945/an.117.015941.

Loring, D.W.; Bowden, S.C. The STROBE statement and neuropsychology: lighting the way toward evidence-based practice. *Clin. Neuropsychol.* **2014**, *28*(4), 556–574. doi: 10.1080/13854046.2012.762552.

Poorolajal, J.; Cheraghi, Z.; Irani, A.D.; Rezaeian, S. Quality of cohort studies reporting post the Strengthening the Reporting of Observational Studies in Epidemiology (STROBE) Statement. *Epidemiol. Health* **2011**, *33*, e2011005. doi: 10.4178/epih/e2011005.

Sharp, M.K.; Hren, D.; Altman, D.G. The STROBE extensions: Considerations for development. *Epidemiology* **2018**, *29*(6), e53–e56. doi: 10.1097/EDE.0000000000000899.

Sharp, M.K.; Bertizzolo, L.; Rius, R.; Wager, E.; Gómez, G.; Hren, D. Using the STROBE statement: survey findings emphasized the role of journals in enforcing reporting guidelines. *J. Clin. Epidemiol.* **2019**, *116*, 26–35. doi: 10.1016/j.jclinepi.2019.07.019. Epub 2019 Aug 6.

Sharp, M.K.; Glonti, K.; Hren, D. Online survey about the STROBE statement highlighted diverging views about its content, purpose, and value. *J. Clin. Epidemiol.* **2020**, *123*, 100–106. doi: 10.1016/j.jclinepi.2020.03.025.

von Elm, E.; Altman, D.G.; Egger, M.; Pocock, S.J.; Gøtzsche, P.C.; Vandenbroucke, J.P.; STROBE Initiative. Strengthening the Reporting of Observational Studies in Epidemiology (STROBE) statement: guidelines for reporting observational studies. *BMJ* **2007**, *335*(7624), 806–808. doi: 10.1136/bmj.39335.541782.AD.

von Elm, E.; Altman, D.G.; Egger, M.; Pocock, S.J.; Gøtzsche, P.C.; Vandenbroucke, J.P.; STROBE Initiative. The Strengthening the Reporting of Observational Studies in Epidemiology (STROBE) statement: guidelines for reporting observational studies. *J. Clin. Epidemiol.* **2008**, *61*(4), 344–349. doi: 10.1016/j.jclinepi.2007.11.008.

von Elm, E.; Altman, D.G.; Egger, M.; Pocock, S.J.; Gøtzsche, P.C.; Vandenbroucke, J.P.; STROBE Initiative. The Strengthening the Reporting of Observational Studies in Epidemiology (STROBE) Statement: guidelines for reporting observational studies. *Int. J. Surg.* **2014**, *12*(12), 1495–1499. doi: 10.1016/j.ijsu.2014.07.013.
